# Phenotype-Oriented Characterization of NSC828786 Identifies Convergent HPN-AMACR-Associated Transcriptomic Signatures in Prostate Adenocarcinoma and Broad-Spectrum Antiproliferative Activity

**DOI:** 10.3390/cells15141314

**Published:** 2026-07-22

**Authors:** Ya-Ting Wen, Rosario Trijuliamos Manalu, Han-Lin Hsu, Yu-Cheng Kuo, Ruey-Shyang Soong, Feng-Cheng Liu, Maryam Rachmawati Sumitra, Sheng-Liang Huang, Shih-Yu Lee, Sung-Ling Tang, I-Chuan Yen, Hong-Jaan Wang, Bashir Lawal, Alexander T. H. Wu, Hsu-Shan Huang

**Affiliations:** 1 Department of Neurosurgery, Wan Fang Hospital, Taipei Medical University, Taipei 11681, Taiwan; 98142@w.tmu.edu.tw; 2 Division of Neurosurgery, Department of Surgery, School of Medicine, College of Medicine, Taipei Medical University, Taipei 11031, Taiwan; 3 Graduate Institute of Cancer Biology and Drug Discovery, College of Medical Science and Technology, Taipei Medical University, Taipei 11031, Taiwan; d621112006@tmu.edu.tw (R.T.M.); maryamrachma60@gmail.com (M.R.S.); 4 Faculty of Pharmacy, National Institute of Science and Technology, Jakarta 12630, Indonesia; 5 Graduate Institute of Clinical Medicine, College of Medicine, Taipei Medical University, Taipei 11031, Taiwan; 94401@w.tmu.edu.tw; 6 Department of Pharmacology, College of Medicine, Taipei Medical University, Taipei 11031, Taiwan; yuchengkuo@tmu.edu.tw; 7 Division of General Surgery, Department of Surgery, Shuang Ho Hospital, Taipei Medical University, New Taipei City 23561, Taiwan; kodlp62@gmail.com; 8 Department of Surgery, School of Medicine, College of Medicine, Taipei Medical University, Taipei 11031, Taiwan; 9 Rheumatology/Immunology and Allergy, Department of Internal Medicine, National Defense Medical University, Taipei 11402, Taiwan; lfc10399@yahoo.com.tw; 10 Graduate Institute of Aerospace and Undersea Medicine, National Defense Medical University, Taipei 11490, Taiwan; angelsapmt@gmail.com (S.-L.H.); leeshihyuno1@mail.ndmctsgh.edu.tw (S.-Y.L.); 11 School of Pharmacy, National Defense Medical University, Taipei 11490, Taiwan; tangling@mail.ndmctsgh.edu.tw (S.-L.T.); yenichuan@mail.ndmctsgh.edu.tw (I.-C.Y.); hongjaan@mail.ndmctsgh.edu.tw (H.-J.W.); 12 UPMC Hillman Cancer Center, University of Pittsburgh, Pittsburgh, PA 15232, USA; bashirlawal12@gmail.com; 13 The Ph.D. Program of Translational Medicine, College of Medical Science and Technology, Taipei Medical University, Taipei 11031, Taiwan; 14 Taipei Heart Institute (THI), Taipei Medical University, Taipei 11031, Taiwan; 15 Ph.D. Program in Biotechnology Research and Development, College of Pharmacy, Taipei Medical University, Taipei 11031, Taiwan

**Keywords:** NSC828786, niclosamide-like salicylanilide, prostate adenocarcinoma, AMACR, HPN, phenotype-oriented analysis

## Abstract

**Highlights:**

**What are the main findings?**

- NSC828786, a niclosamide-like salicylanilide derivative, demonstrated broad-spectrum antiproliferative activity across the NCI-60 cancer cell panel without evidence of receptor subtype-selective activity.
- Cross-cohort transcriptomic analyses consistently identified HPN and AMACR as candidate tumor-associated genes across independent prostate adenocarcinoma cohorts.

**What are the implications of the main findings?**

- Phenotype-oriented integration of pharmacological and transcriptomic analyses provides an exploratory framework for prioritizing candidate molecular contexts beyond conventional single-target drug discovery.
- NSC828786 represents an exploratory salicylanilide lead requiring direct target engagement studies, mechanistic validation, pharmacokinetic evaluation, and in vivo validation before therapeutic implications can be established.

**Abstract:**

Prostate cancer remains a major cause of cancer-related mortality, and new therapeutic strategies are needed for advanced disease. Niclosamide and related salicylanilide compounds have emerged as multitarget anticancer agents, but the molecular contexts associated with their activity remain incompletely understood. Here, we applied a phenotype-oriented integrative framework to characterize the molecular context and phenotypic activity of NSC828786, a niclosamide-like salicylanilide derivative. Cross-cohort transcriptomic analyses identified AMACR (alpha-methylacyl-CoA racemase) and HPN (hepsin) as consistently upregulated genes in independent prostate adenocarcinoma cohorts. NCI-60 profiling demonstrated broad-spectrum low-micromolar antiproliferative activity, including AR-negative prostate cancer and breast cancer cell lines spanning multiple receptor subtypes; however, quantitative ranking did not support preferential receptor subtype selectivity. CellMiner COMPARE analysis showed no significant correlation between baseline AMACR or HPN expression and NSC828786 sensitivity. Structure-based analyses supported computational compatibility of NSC828786 with predicted HPN- and AMACR-associated binding regions, while zebrafish assays showed no overt developmental abnormalities at concentrations ≤ 5 μM. These findings identify NSC828786 as a phenotypically active salicylanilide derivative and position HPN and AMACR as exploratory candidate molecular associations warranting further mechanistic and target engagement studies.

## 1. Introduction

Prostate cancer remains one of the most frequently diagnosed malignancies and a leading cause of cancer-related mortality among men worldwide [1]. Although androgen deprivation therapy (ADT) and next-generation androgen receptor (AR) pathway inhibitors have substantially improved clinical outcomes, therapeutic resistance remains a major obstacle in advanced prostate cancer [2]. Increasing evidence suggests that disease progression is influenced not only by alterations in canonical AR signaling but also by broader changes in tumor metabolism, extracellular remodeling, and microenvironmental adaptation [3]. However, the molecular contexts integrating these biological processes remain incompletely understood.

Metabolic remodeling and extracellular proteolysis have emerged as important features of prostate cancer progression [4]. Alterations in lipid and fatty acid metabolism support tumor bioenergetics, redox homeostasis, and anabolic demands, whereas extracellular protease activity promotes matrix remodeling, epithelial plasticity, and tumor invasion [5]. Although these processes have traditionally been investigated independently, accumulating evidence suggests that they may converge during tumor progression and adaptation [6]. Identifying transcriptomic signatures that reproducibly capture these biological features across independent patient cohorts may facilitate the prioritization of candidate molecular contexts relevant to prostate cancer biology [7].

Salicylanilide-based compounds have recently attracted considerable attention because of their broad pharmacological activities in cancer [8]. Among these, niclosamide, an FDA-approved anthelmintic agent, has demonstrated anticancer activity through modulation of multiple signaling pathways, including AR-V7, Wnt/β-catenin, STAT3, mTOR, NF-κB, and mitochondrial oxidative phosphorylation [9,10,11]. In prostate cancer models, niclosamide has been reported to suppress AR-V7 expression, enhance the activity of AR-targeted therapies, and inhibit the growth of treatment-resistant tumor cells [12]. Despite these encouraging findings, the broader molecular contexts associated with salicylanilide responsiveness remain incompletely characterized.

NSC828786 is a structurally modified salicylanilide derivative developed through our previous scaffold optimization studies [13]. The compound retains the characteristic salicylanilide pharmacophore, including a phenolic hydroxyl group, an amide linker, and halogen-substituted aromatic rings. Previous studies demonstrated that NSC828786 suppresses tumor growth and induces endoplasmic reticulum stress-associated autophagy in prostate cancer models, suggesting that its biological activity may extend beyond modulation of a single signaling pathway [14]. These observations prompted us to investigate the broader molecular context associated with its phenotypic activity.

Alpha-methylacyl-CoA racemase (AMACR) is a peroxisomal and mitochondrial enzyme that catalyzes the stereoconversion of branched-chain fatty acid and bile acid intermediates during β-oxidation and is among the most extensively validated diagnostic biomarkers for prostate adenocarcinoma [15,16]. Hepsin (HPN) is a type II transmembrane serine protease involved in extracellular proteolysis through activation of substrates such as pro-hepatocyte growth factor (pro-HGF), pro-urokinase plasminogen activator (pro-uPA), and pro-macrophage stimulating protein (pro-MSP), thereby contributing to epithelial remodeling and tumor invasion [17,18,19]. Although AMACR and HPN have each been extensively investigated in prostate cancer, they have rarely been considered together as convergent transcriptomic features representing complementary metabolic and extracellular remodeling functions.

In the present study, we applied a phenotype-oriented integrative framework to characterize the molecular context associated with the phenotypic activity of NSC828786. By integrating cross-cohort transcriptomic analyses, functional enrichment, network analysis, structure-based computational modeling, NCI-60 pharmacological profiling [20], and zebrafish embryo tolerability assessment [21], we identified HPN and AMACR as convergent transcriptomic signatures consistently upregulated across independent prostate adenocarcinoma cohorts and evaluated their potential association with the pharmacological profile of NSC828786. Rather than establishing HPN or AMACR as validated molecular targets, this study positions these genes as exploratory candidate molecular associations identified through transcriptomic integration and computational analyses. Together, our findings provide a hypothesis-generating framework for future mechanistic, target engagement, and functional validation studies of NSC828786.

## 2. Materials and Methods

### 2.1. Compounds and Reagents

NSC828786, also designated as LCC03 (C_19_H_11_F_4_NO_2_), is a structurally modified salicylanilide derivative synthesized in-house as previously described [13]. Compound purity was confirmed to be >98% by high-performance liquid chromatography (HPLC), and structural identity was verified by ^1^H and ^13^C nuclear magnetic resonance spectroscopy. The compound was dissolved in dimethyl sulfoxide (DMSO) to prepare stock solutions and stored at −20 °C until use. For cell-based assays, working solutions were freshly diluted in culture medium, and the final DMSO concentration did not exceed 0.1% (*v*/*v*). Vehicle controls contained equivalent concentrations of DMSO.

### 2.2. Transcriptomic and Bioinformatic Analyses

Publicly available prostate and breast cancer transcriptomic datasets were retrieved from the Gene Expression Omnibus (GEO) database and analyzed to identify differentially expressed genes between tumor and corresponding normal tissues [22]. Differential expression analyses were performed using the limma package in R (version 4.3.2) following background correction, log2 transformation, and normalization as described in the Supplementary Methods. Genes with an absolute log2 fold change (|log2FC|) > 1 and a Benjamini–Hochberg false discovery rate (FDR)-adjusted *p* value < 0.05 were considered significantly dysregulated [23]. Cross-cohort intersection analysis was performed to identify genes consistently dysregulated across independent prostate adenocarcinoma cohorts. Functional enrichment, protein–protein interaction, cancer hallmark, and pan-cancer expression analyses were performed using STRING, WebGestalt, Metascape, GEPIA2, and CHAT [24]. Genomic alterations, transcriptomic correlations, and protein expression patterns were evaluated using The Cancer Genome Atlas (TCGA), cBioPortal, and the Human Protein Atlas (HPA). Clinical and pathological information available for each GEO dataset, including pathological stage, histological grade, receptor status (where applicable), treatment information, and other publicly available metadata, was manually curated from the original GEO records and is summarized in Supplementary Table S1. Variables unavailable in the original datasets are explicitly designated as “NR” (not reported). Because the analyzed datasets primarily compared tumor tissues with corresponding normal tissues, the transcriptomic analyses were interpreted as identifying tumor-associated molecular signatures rather than molecular features specific to castration-resistant disease, androgen receptor status, or breast cancer receptor subtypes. Detailed dataset accession numbers, preprocessing procedures, normalization methods, and statistical parameters are provided in the Supplementary Methods.

### 2.3. NCI-60 Pharmacological Profiling

The antiproliferative activity of NSC828786 was evaluated using the NCI-60 human tumor cell line panel available through the National Cancer Institute Developmental Therapeutics Program (NCI-DTP). Growth inhibitory activity was determined using the standardized five-dose sulforhodamine B (SRB) assay, from which the pharmacological response parameters GI_50_ (50% growth inhibition), TGI (total growth inhibition), and LC_50_ (50% lethal concentration) were calculated according to the established NCI-DTP protocol [20]. These pharmacological parameters were retrieved directly from the publicly available NCI-DTP database. For descriptive comparison, single-dose (10 μM) growth inhibition data were also retrieved from the NCI-DTP database and used to illustrate the relative growth response of selected prostate and breast cancer cell lines. These single-dose screening results are presented separately from the five-dose GI_50_ analyses. Pattern similarity analyses were performed using the CellMiner COMPARE platform to compare the pharmacological response profile of NSC828786 with those of selected reference compounds, including niclosamide and clinically relevant anticancer agents available in the NCI-60 database. In addition, Pearson correlation analyses were performed using the CellMiner COMPARE gene expression module to evaluate the association between baseline AMACR and HPN transcript expression and NSC828786 sensitivity across the NCI-60 panel. Correlation coefficients (r), corresponding *p* values, and Benjamini–Hochberg false discovery rate (FDR)-adjusted *p* values were calculated and are summarized in Supplementary Table S2. Because all pharmacological data were obtained from the publicly accessible NCI-DTP resource, information regarding individual positive control compounds used during compound screening is not available in the public database.

### 2.4. Structure-Based Computational Analysis

Structure-based computational analyses were performed to evaluate the structural compatibility of NSC828786 with HPN- and AMACR-associated molecular environments. Protein structures were obtained from the Protein Data Bank (PDB) and the AlphaFold Protein Structure Database [25]. Ligand structures were generated and energy-minimized prior to docking simulations. Molecular docking analyses were performed using AutoDock Vina v1.2.5 [26], with docking grids centered on the catalytic or substrate-associated regions of each target protein. Predicted binding poses and ligand–protein interactions were analyzed and visualized using molecular modeling software. Molecular dynamics (MD) simulations were subsequently performed to evaluate the conformational stability of ligand-bound complexes, and root-mean-square fluctuation (RMSF) analyses were conducted to assess residue-level flexibility throughout the simulation period [27]. The colored RMSF profiles represent independent ligand-bound simulations for NSC828786 and the reference compounds included in the comparative analyses, as described in the corresponding figure legend. In silico developability analyses, including physicochemical property prediction, aqueous solubility, lipophilicity, absorption-related descriptors, blood–brain barrier permeability, and toxicity-associated parameters, were performed using SwissADME, pkCSM, and BBB Predictor accessed on 20 April 2026 [28]. Complete docking protocols, molecular dynamics simulation settings, and developability prediction parameters are provided in the Supplementary Methods.

### 2.5. Zebrafish Embryo Developmental Tolerability Assay

Wild-type zebrafish (Danio rerio) embryos were maintained under standard laboratory conditions at the Core Laboratory of Zebrafish, Taipei Medical University, in accordance with institutional animal care guidelines and standard zebrafish husbandry procedures [29]. Embryos derived from a single breeding pair were randomly allocated to control and treatment groups. Compound exposure was initiated at 4 h post-fertilization (hpf), and embryos were incubated in E3 embryo medium containing NSC828786 (0, 5, 10, or 15 μM) until 96 hpf. The final dimethyl sulfoxide (DMSO) concentration was maintained at 0.1% (*v*/*v*) in all treatment groups. Morphological development, survival, hatching, body length, yolk sac area, pericardial cavity area, and eye area were evaluated at predefined developmental stages using stereomicroscopy and ImageJ v1.54d-assisted image analysis. Morphometric measurements were performed using coded image files to minimize observer bias. Nominal exposure concentrations were used throughout the study, and no visible compound precipitation was observed under the experimental conditions. Complete experimental procedures, embryo numbers, exposure conditions, statistical analyses, and morphometric assessment protocols are provided in the Supplementary Methods.

### 2.6. Statistical Analysis

Statistical analyses were performed using R (version 4.3.2; R Foundation for Statistical Computing, Vienna, Austria) and GraphPad Prism (version 10.0; GraphPad Software, San Diego, CA, USA). Differential gene expression analyses were conducted using the limma package with Benjamini–Hochberg false discovery rate (FDR) correction for multiple testing [30]. Comparisons between two groups were performed using Student’s *t*-test or the Wilcoxon rank-sum test, as appropriate according to data distribution. Comparisons among multiple groups were performed using one-way analysis of variance (ANOVA) followed by Dunnett’s multiple comparisons test, where each treatment group was compared with the corresponding control group. Correlation analyses were performed using Pearson’s or Spearman’s correlation coefficients, as appropriate. Data are presented as mean ± standard deviation (SD) unless otherwise specified. All statistical tests were two-sided, and *p* values < 0.05 were considered statistically significant. Additional statistical procedures, including endpoint-specific analyses for the zebrafish embryo assays and bioinformatic analyses, are described in the Supplementary Methods.

## 3. Results

### 3.1. Broad-Spectrum Antiproliferative Activity of NSC828786 Across the NCI-60 Cancer Cell Panel

**Figure 1:**
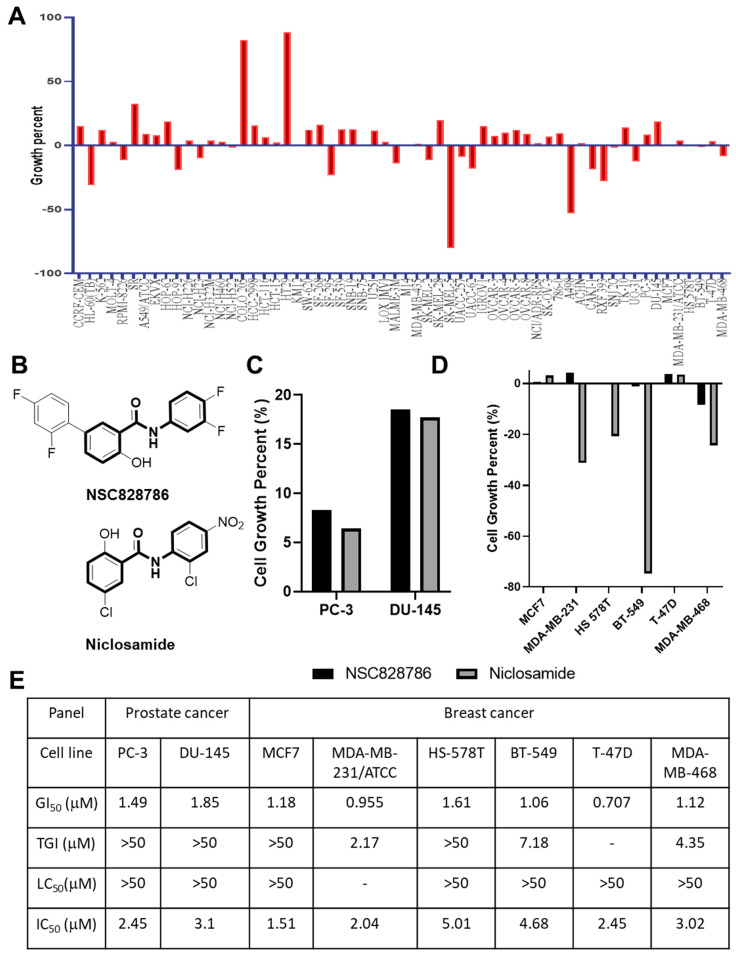
Broad−spectrum antiproliferative activity of NSC828786 across the NCI-60 human cancer cell line panel. (**A**) Single-dose (10 μM) growth response profile of NSC828786 across the NCI-60 human cancer cell line panel obtained from the NCI-DTP. Positive values indicate growth inhibition, whereas negative values indicate net cell growth relative to untreated controls. (**B**) Chemical structures of NSC828786 and niclosamide, illustrating their shared salicylanilide scaffold. (**C**,**D**) Pharmacological response profiling and comparative analyses of NSC828786 and niclosamide across representative prostate and breast cancer models. (**E**) Summary of the five-dose pharmacological response parameters of NSC828786 in representative prostate and breast cancer cell lines, including GI_50_, TGI, LC_50_, and IC_50_ values.

NCI-60 profiling demonstrated preferential sensitivity of AR-negative prostate cancer cell lines to NSC828786 exposure, with additional activity observed in breast cancer models used as hormone-independent epithelial comparators (Figure 1). PC-3 and DU-145 prostate cancer cells exhibited low-micromolar GI50 values, with comparable or greater sensitivity observed in several breast cancer lines irrespective of receptor subtype (Supplementary Table S2). Across responsive cell lines, TGI and LC_50_ values generally exceeded GI_50_ values, indicating a predominantly cytostatic pharmacological profile. Dose-dependent growth inhibition was observed in both prostate and breast cancer models (Figure 1E). PC-3 cells showed greater responsiveness than DU-145 cells, while MDA-MB-231 and BT-549 were among the more sensitive breast cancer comparators. COMPARE analysis demonstrated limited similarity to conventional cytotoxic agents, suggesting a distinct response pattern. Collectively, these findings identify NSC828786 as a phenotypically active salicylanilide derivative in hormone-independent cancer models. We note that the NCI-60 panel includes only AR-negative prostate cancer cell lines (PC-3, DU-145), precluding any direct comparison with AR-positive models within this dataset; independent confirmation in AR-positive prostate cancer cell lines (e.g., LNCaP, VCaP, 22Rv1) is required to determine whether NSC828786 activity differs by androgen receptor status (Supplementary Table S2). Collectively, these findings identify NSC828786 as a phenotypically active salicylanilide derivative with broad antiproliferative activity across the NCI-60 panel, without evidence supporting subtype-selective activity based on the present dataset.

### 3.2. Cross-Cohort Transcriptomic Analyses Identify Convergent HPN and AMACR Upregulation

To identify transcriptomic signatures associated with prostate and breast cancers, we analyzed eight independent GEO microarray datasets comprising five prostate adenocarcinoma cohorts and three breast cancer cohorts, each including tumor and matched normal tissues (Supplementary Table S1; Supplementary Methods) [31]. Differential expression analyses were performed using the limma package with stringent filtering (|log2FC| > 1 and Benjamini–Hochberg FDR-adjusted *p* < 0.05) [32]. Volcano plots for individual datasets are shown in Supplementary Figure S1, and complete lists of differentially expressed genes are provided. Cross-cohort intersection analysis identified HPN and AMACR as consistently upregulated genes across the prostate adenocarcinoma cohorts. Similar enrichment patterns were observed in the breast cancer cohorts. Pan-cancer analyses using GEPIA2 and CHAT demonstrated preferential enrichment of both genes in prostate and breast cancers relative to many other tumor types [33]. Validation using TCGA and TNMplot datasets confirmed significantly increased HPN and AMACR expression in tumor tissues compared with corresponding normal tissues. Stage-stratified analyses showed persistent expression across disease stages without substantial stage-dependent variation. Transcriptomic profiling across the NCI-60 panel further demonstrated above-average expression of HPN and AMACR in the AR-negative prostate cancer cell lines PC-3 and DU-145. CellMiner COMPARE analysis showed that baseline AMACR and HPN expression was not significantly correlated with NSC828786 sensitivity (AMACR: r = 0.096, *p* = 0.477; HPN: r = 0.112, *p* = 0.405; Benjamini–Hochberg FDR-adjusted *p* = 0.477 for both). These findings indicate that baseline AMACR and HPN transcript expression alone does not predict NSC828786 antiproliferative activity across the NCI-60 panel. Accordingly, the association between NSC828786 and HPN/AMACR identified through differential-expression and computational analyses should be interpreted as an exploratory candidate molecular association rather than evidence of a functional or mechanistic relationship.

### 3.3. HPN- and AMACR-Associated Interaction Networks Are Enriched in Metabolic and Proteolytic Biological Processes

Protein interaction and pathway enrichment analyses were performed to further characterize the biological context associated with the consistent upregulation of HPN and AMACR across independent prostate adenocarcinoma cohorts. STRING protein–protein interaction analysis demonstrated that both proteins were embedded within interconnected molecular networks enriched for lipid metabolism, extracellular matrix organization, proteolytic regulation, and epithelial signaling pathways (Supplementary Figure S2A) [34]. Gene Ontology enrichment analysis further identified significant enrichment of biological processes related to extracellular matrix organization, proteolysis, regulation of peptidase activity, and lipid metabolic processes (Supplementary Figure S2B) [35]. Complementary Hallmark and pathway enrichment analyses identified significant associations with metabolic reprogramming, extracellular matrix remodeling, angiogenesis, inflammatory signaling, and pathways involved in cellular proliferation and survival (Supplementary Figure S2C,D) [36]. These enriched pathways are consistent with biological processes frequently associated with prostate tumor progression; however, because the enrichment analyses were derived from tumor-versus-normal differential expression datasets, they should be interpreted as exploratory transcriptomic associations rather than evidence of therapy resistance, androgen receptor independence, or causal molecular mechanisms. Collectively, these findings position HPN and AMACR within interconnected metabolic and proteolytic molecular networks that may contribute to the biological landscape of prostate adenocarcinoma, providing a systems-level framework for subsequent computational analyses while generating hypotheses for future mechanistic investigation.

### 3.4. Genomic and Protein-Level Characterization of HPN and AMACR

**Figure 2:**
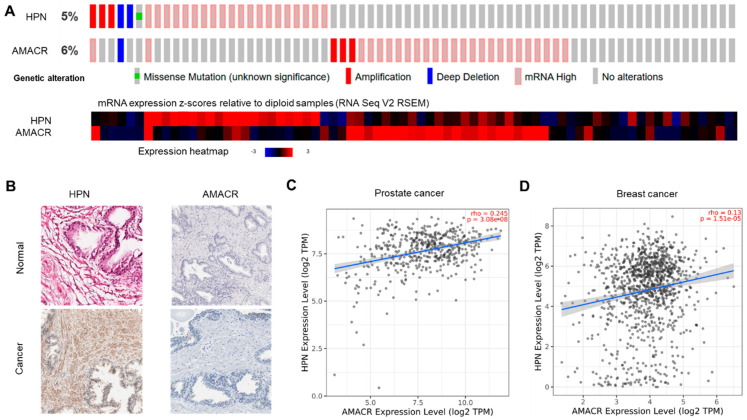
Genomic, transcriptomic, and protein−level characterization of HPN and AMACR in prostate adenocarcinoma. (**A**) cBioPortal OncoPrint showing genomic alterations and corresponding mRNA expression patterns of HPN and AMACR in the TCGA prostate adenocarcinoma (PRAD) cohort. Alterations consist predominantly of gene amplification and increased mRNA expression, whereas coding mutations are infrequent. (**B**) Representative immunohistochemical staining images from the Human Protein Atlas showing HPN and AMACR protein expression in normal prostate tissue and prostate adenocarcinoma. (**C**) Correlation between HPN and AMACR transcript expression in the TCGA prostate adenocarcinoma cohort. (**D**) Correlation between HPN and AMACR transcript expression in the TCGA breast cancer cohort. Expression values are presented as log_2_ TPM, and correlation coefficients (Spearman’s ρ) and corresponding *p* values are shown in each panel.

Genomic and protein-level characteristics of HPN and AMACR were further investigated using publicly available prostate cancer datasets and protein expression resources (Figure 2). Analysis of the TCGA prostate adenocarcinoma (PRAD) cohort through cBioPortal demonstrated relatively low overall alteration frequencies for HPN (5%) and AMACR (6%), with genomic alterations consisting predominantly of gene amplification and mRNA upregulation rather than recurrent coding mutations (Figure 2A) [37]. Because the TCGA-PRAD cohort primarily comprises localized primary prostate adenocarcinoma, these findings should not be interpreted as representing castration-resistant or androgen receptor-independent disease. Representative immunohistochemical images obtained from the Human Protein Atlas demonstrated stronger HPN and AMACR protein staining in prostate adenocarcinoma tissues than in corresponding normal prostate tissues (Figure 2B), consistent with the transcriptomic observations. Correlation analyses further demonstrated significant positive associations between HPN and AMACR transcript expression in both the TCGA prostate adenocarcinoma cohort (ρ = 0.245, *p* = 3.08 × 10^−8^; Figure 2C) and the TCGA breast cancer cohort (ρ = 0.130, *p* = 1.15 × 10^−5^; Figure 2D). Additional cBioPortal analyses identified co-occurring alterations involving several genes frequently altered in prostate cancer, including TMPRSS2, ERG, PTEN, TP53, and AR (Supplementary Figure S3). Collectively, these independent genomic, transcriptomic, and protein-expression datasets consistently demonstrate increased HPN and AMACR expression in prostate adenocarcinoma. However, these descriptive analyses do not establish functional interactions between HPN, AMACR, and NSC828786, nor do they demonstrate associations with castration resistance, androgen receptor independence, or therapeutic responsiveness. Instead, these observations provide an exploratory molecular context for subsequent computational analyses and future mechanistic investigation.

### 3.5. Structure-Based Analyses Support Structural Compatibility of NSC828786 with Predicted HPN and AMACR Binding Regions

**Figure 3:**
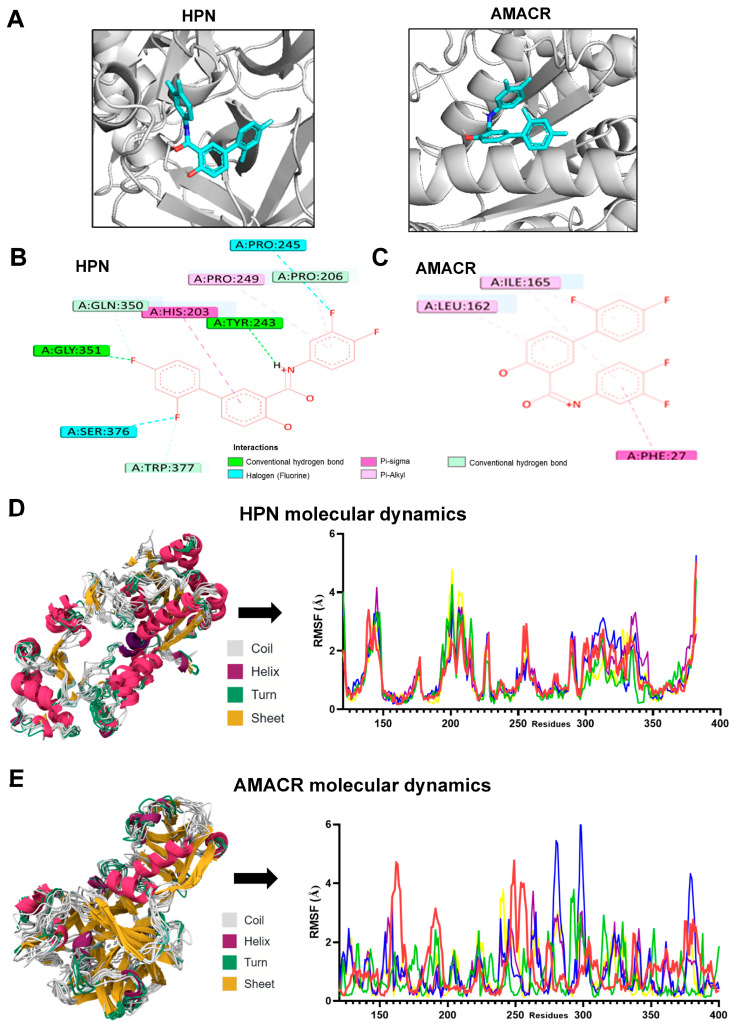
Structure−based docking and molecular dynamics analyses of NSC828786 in HPN and AMACR. (**A**) Predicted docking poses of NSC828786 within the putative ligand-binding regions of HPN (**left**) and AMACR (**right**). The protein structures are shown in cartoon representation, and NSC828786 is displayed as cyan sticks. (**B**) Two-dimensional interaction diagrams illustrating the predicted interactions between NSC828786 and residues surrounding the HPN catalytic pocket. (**C**). Two-dimensional interaction diagrams illustrating the predicted interactions between NSC828786 and residues within the AMACR substrate-associated binding region. (**D**) Molecular dynamics trajectory superposition (**left**) and residue-wise root-mean-square fluctuation (RMSF) analysis (**right**) of HPN complexes. RMSF profiles compare simulations performed with NSC828786 (red), apalutamide (green), enzalutamide (blue), niclosamide (purple), and honokiol (yellow). Differences among the RMSF profiles reflect ligand-dependent local protein flexibility throughout the simulation. (**E**) Molecular dynamics trajectory superposition (**left**) and residue-wise RMSF analysis (**right**) of AMACR complexes. RMSF profiles compare simulations performed with NSC828786 (red), apalutamide (green), enzalutamide (blue), niclosamide (purple), and honokiol (yellow). These analyses provide a comparative assessment of ligand-associated conformational flexibility and do not constitute direct evidence of target engagement or biochemical inhibition.

Structure-based molecular docking was performed to explore whether NSC828786 could adopt energetically favorable binding conformations within the predicted ligand-binding regions of HPN and AMACR. Docking simulations predicted that NSC828786 occupied the HPN catalytic pocket and the substrate-associated binding region of AMACR, generating plausible binding poses with favorable docking scores (Figure 3A–C; Supplementary Table S2). In the predicted HPN complex, NSC828786 formed hydrogen-bonding and hydrophobic interactions with residues surrounding the catalytic region, whereas binding within AMACR was primarily stabilized through hydrophobic contacts within the substrate-associated cleft. These predicted interaction patterns were consistent with the structural characteristics of the respective ligand-binding environments. To further evaluate the dynamic behavior of the predicted complexes, molecular dynamics simulations were performed and compared with those of apalutamide, enzalutamide, niclosamide, and honokiol. Representative trajectory superposition demonstrated overall structural stability of both ligand-bound complexes throughout the simulation period (Figure 3D,E, left panels). Residue-wise RMSF analyses showed ligand-dependent differences in local protein flexibility, with NSC828786 exhibiting RMSF profiles that were generally comparable to those of the reference compounds, although several localized regions displayed modestly increased fluctuations relative to the comparator ligands (Figure 3D,E, right panels). These computational analyses suggest that NSC828786 is structurally compatible with predicted ligand-binding regions of HPN and AMACR. However, docking poses and molecular dynamics simulations represent in silico structural predictions only and do not establish direct biochemical inhibition, target engagement, or a causal contribution of either protein to the antiproliferative activity of NSC828786. Experimental validation using orthogonal target engagement approaches (e.g., CETSA, DARTS, biochemical assays, or genetic perturbation) will be required to determine whether these computationally predicted interactions are biologically relevant.

### 3.6. In Silico Physicochemical and Developability Assessment of NSC828786

In silico developability analyses were performed using SwissADME, pkCSM, and BBB Predictor, and the corresponding results are summarized in Supplementary Table S3 and Supplementary Figure S4 [38]. NSC828786 exhibited physicochemical properties generally compatible with a small-molecule scaffold, including a molecular weight of 361.29 Da, limited conformational flexibility, and compliance with several commonly used drug-likeness criteria [39]. However, the compound also demonstrated a relatively high predicted lipophilicity (consensus LogP = 5.20) together with poor predicted aqueous solubility (predicted LogS values ranging from −6.08 to −7.93), indicating potential formulation and developability challenges that should be considered during future preclinical optimization. Computational toxicity predictions identified potential safety liabilities, including predicted probabilities of hERG channel blockade and hepatotoxicity, although these predictions were generated using machine learning–based computational models and require experimental validation [40]. Accordingly, these results should be interpreted as preliminary in silico risk assessments rather than definitive indicators of compound safety. Blood–brain barrier permeability was evaluated using a dedicated BBB prediction model rather than skin permeation (logKp) parameters. Because BBB prediction models remain computational estimates, no conclusions regarding central nervous system exposure or distribution can be drawn from the present analyses alone. Collectively, these findings suggest that NSC828786 possesses several favorable drug-like molecular characteristics while also exhibiting predicted developability challenges, particularly with respect to aqueous solubility and potential safety liabilities. Therefore, comprehensive pharmacokinetic, formulation, and toxicological studies will be required before the therapeutic potential of NSC828786 can be fully evaluated.

### 3.7. Zebrafish Developmental Tolerability Assessment

**Figure 4:**
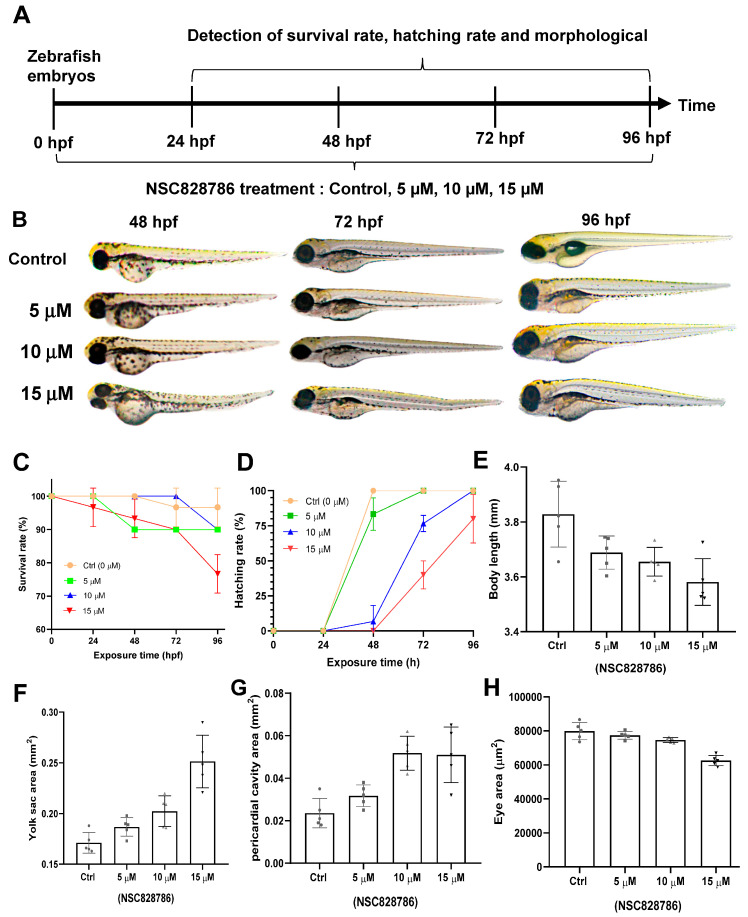
Developmental tolerability assessment of NSC828786 in zebrafish embryos. (**A**) Experimental design illustrating zebrafish embryo exposure to NSC828786 (0, 5, 10, and 15 μM) from 4 to 96 h post-fertilization (hpf). (**B**) Representative bright-field images of zebrafish embryos at 48, 72, and 96 hpf following NSC828786 exposure. Scale bars = 200 μm. (**C**) Survival rates of zebrafish embryos during NSC828786 exposure. (**D**) Hatching rates of zebrafish embryos during development. (**E**–**H**) Quantitative morphometric analyses at 96 hpf, including body length (**E**), yolk sac area (**F**), pericardial cavity area (**G**), and eye area (**H**). Data are presented as mean ± SD. Morphometric analyses were performed on individual embryos (*n* = 5 per treatment group).

To obtain a preliminary organism-level assessment of developmental tolerability, zebrafish embryos were exposed to NSC828786 (0, 5, 10, or 15 μM) from 4 to 96 h post-fertilization (hpf) using nominal exposure concentrations (Figure 4A). No visible precipitation of NSC828786 was observed during the exposure period under the experimental conditions used. Representative morphological observations demonstrated that embryos exposed to 5 μM NSC828786 developed normally and were comparable to vehicle-treated controls throughout embryogenesis (Figure 4B). In contrast, embryos exposed to 10 and 15 μM exhibited concentration-dependent developmental abnormalities, accompanied by reduced survival and delayed hatching during later developmental stages (Figure 4C,D) [21]. Quantitative morphometric analyses performed at 96 hpf further demonstrated dose-dependent reductions in body length, together with increased yolk sac area and pericardial cavity area at higher exposure concentrations (Figure 4E–G). Eye area was significantly reduced only in embryos exposed to 15 μM NSC828786 (Figure 4H). Overall, these observations indicate that developmental effects became apparent at concentrations ≥ 10 μM, whereas 5 μM produced no overt developmental abnormalities under the present experimental conditions. Because these experiments were performed using embryos derived from a single breeding event, the findings should be interpreted as preliminary developmental tolerability data. Confirmation using independent biological replicates from multiple clutches will be required to further establish the developmental safety profile of NSC828786. For comparison, niclosamide has previously been reported to be among the most potent developmental toxicants identified in zebrafish embryo models, with exposure to 10 μM during early gastrulation (5–25 hpf) resulting in complete embryonic lethality through disruption of epiboly [41]. These published findings provide a reference for interpreting the developmental tolerability of NSC828786 but should not be considered a direct head-to-head comparison because the exposure windows and experimental conditions differed between studies.

## 4. Discussion

In this study, we applied a phenotype-oriented integrative framework combining NCI-60 pharmacological profiling, cross-cohort transcriptomic analyses, publicly available cancer resources, and structure-based computational modeling to characterize the biological context associated with the antiproliferative activity of NSC828786. NSC828786 exhibited broad-spectrum low-micromolar antiproliferative activity across the NCI-60 panel, including the AR-negative prostate cancer cell lines PC-3 and DU-145 as well as breast cancer cell lines representing multiple receptor subtypes. Consistent with the revised pharmacological analyses, the present data do not support preferential activity toward AR-negative prostate cancer or triple-negative breast cancer. Rather, the results position NSC828786 as a phenotypically active salicylanilide-derived small molecule whose activity can be interpreted within an exploratory transcriptomic and computational framework.

Cross-cohort transcriptomic analyses consistently identified HPN and AMACR as genes upregulated in independent prostate adenocarcinoma cohorts, with additional support from TCGA-derived datasets, Human Protein Atlas immunohistochemistry, and NCI-60 transcriptomic profiles [42]. AMACR is a key enzyme involved in branched-chain fatty acid metabolism [43], whereas HPN encodes a type II transmembrane serine protease implicated in extracellular matrix remodeling and epithelial invasion [44]. Although these genes represent well-established prostate cancer–associated biomarkers, our systems-level analyses suggest that they occupy complementary metabolic and proteolytic biological contexts. Importantly, because the transcriptomic analyses were derived from tumor-versus-normal comparisons, they do not establish associations with castration resistance, androgen receptor independence, lineage plasticity, or therapeutic resistance, and these relationships will require validation in receptor-defined and resistance-stratified clinical cohorts.

Functional enrichment analyses further associated HPN- and AMACR-related gene networks with extracellular matrix organization, proteolysis, lipid metabolism, inflammatory signaling, angiogenesis, and cell survival–related pathways. These biological processes have individually been implicated in tumor progression and adaptive remodeling in previous studies; however, the present enrichment analyses should be interpreted as exploratory transcriptomic associations rather than evidence of causal biological mechanisms or therapy-resistant molecular programs. Likewise, this study did not directly evaluate immune-cell infiltration, immune checkpoint signaling, T-cell dysfunction, or immunotherapy responsiveness. Consequently, immune-related processes identified by pathway enrichment provide biological context for future investigation rather than mechanistic evidence of NSC828786 action.

Pharmacologically, NSC828786 displayed a characteristic separation between GI_50_ and LC_50_ values, suggesting a predominantly cytostatic rather than cytotoxic activity profile. Molecular docking and molecular dynamics simulations demonstrated that NSC828786 could adopt energetically favorable conformations within predicted ligand-binding regions of HPN and AMACR, providing a structural framework for exploring potential molecular interactions. Nevertheless, these computational analyses do not demonstrate direct biochemical inhibition, target engagement, or functional involvement of either protein in mediating NSC828786 responsiveness. Definitive validation will require orthogonal experimental approaches, including genetic perturbation, biochemical inhibition assays, and target engagement techniques such as CETSA, DARTS, or thermal proteome profiling.

Computational developability analyses indicated that NSC828786 possesses several favorable small-molecule characteristics while also revealing predicted developability challenges, particularly high lipophilicity, poor predicted aqueous solubility, and computationally predicted safety liabilities that require experimental verification. Zebrafish embryo assays provided preliminary organism-level tolerability data, with no overt developmental abnormalities observed at 5 μM and concentration-dependent developmental effects becoming evident at 10–15 μM. Because these experiments were conducted using nominal exposure concentrations and embryos derived from a single breeding event, the findings should be regarded as preliminary developmental tolerability data requiring confirmation in independent biological replicates together with comprehensive pharmacokinetic and toxicological studies in mammalian systems.

Several limitations should be acknowledged. First, this study establishes systems-level molecular associations rather than direct target validation. Second, the transcriptomic analyses integrate publicly available datasets that differ in experimental platform, cohort composition, and availability of clinical annotations. Third, molecular docking and molecular dynamics simulations generate structure-based hypotheses that require biochemical confirmation. Fourth, the antiproliferative effects of NSC828786 may reflect broader cellular stress responses or adaptive signaling networks rather than inhibition of a single molecular target. Finally, the zebrafish experiments provide only an initial assessment of developmental tolerability and cannot substitute for formal pharmacokinetic, toxicological, or efficacy studies in mammalian models.

Collectively, this study provides a phenotype-oriented and hypothesis-generating framework for characterizing NSC828786 as a salicylanilide-derived small molecule with broad-spectrum antiproliferative activity. By integrating pharmacological profiling, transcriptomic analyses, and structure-based computational modeling, we identified exploratory molecular associations involving HPN and AMACR that warrant further investigation. Future studies incorporating direct target engagement assays, pathway perturbation experiments, pharmacokinetic characterization, and mammalian efficacy models will be required to determine the biological relevance and therapeutic potential of these candidate molecular contexts.

## 5. Conclusions

In this study, we applied a phenotype-oriented integrative framework combining NCI-60 pharmacological profiling, cross-cohort transcriptomic analyses, publicly available cancer datasets, and structure-based computational modeling to characterize the biological context associated with the antiproliferative activity of NSC828786. NSC828786 demonstrated broad-spectrum low-micromolar antiproliferative activity across the NCI-60 panel, including the AR-negative prostate cancer cell lines PC-3 and DU-145 as well as breast cancer cell lines representing multiple receptor subtypes, without evidence supporting subtype-selective activity. Integrative transcriptomic analyses consistently identified HPN and AMACR as convergently upregulated molecular features in independent prostate adenocarcinoma cohorts, while structure-based computational analyses suggested that NSC828786 is structurally compatible with predicted ligand-binding regions of both proteins. However, these observations represent exploratory molecular associations and computational predictions rather than evidence of direct target engagement, biochemical inhibition, or a causal mechanistic relationship. Collectively, this study provides a hypothesis-generating framework linking phenotypic drug activity with transcriptomic and computational analyses, positioning NSC828786 as a salicylanilide-derived lead compound for further investigation rather than as a validated dual-target inhibitor. Future studies incorporating direct target engagement assays, genetic and biochemical perturbation, pharmacokinetic characterization, and efficacy evaluation in mammalian models will be essential to determine the biological relevance, therapeutic mechanism, and translational potential of NSC828786.

## Data Availability

The datasets used and analyzed during the current study are available from the corresponding author upon reasonable request.

## Supplementary Materials

The following supporting information can be downloaded at: https://www.mdpi.com/article/10.3390/cells15141314/s1.
