# Supplementary material for: Phenotype-Oriented Characterization of NSC828786 Identifies Convergent HPN-AMACR-Associated Transcriptomic Signatures in Prostate Adenocarcinoma and Broad-Spectrum Antiproliferative Activity
Source: Cells. 2026 Jul 22;15(14):1314. doi: 10.3390/cells15141314 (PMC13406622; doi:10.3390/cells15141314)
Supplement: Supplementary file 1 [file cells-15-01314-s001.zip › Supplementary Fig. S3_20260630_final_revised.pdf]

Supplementary Figure S3

A

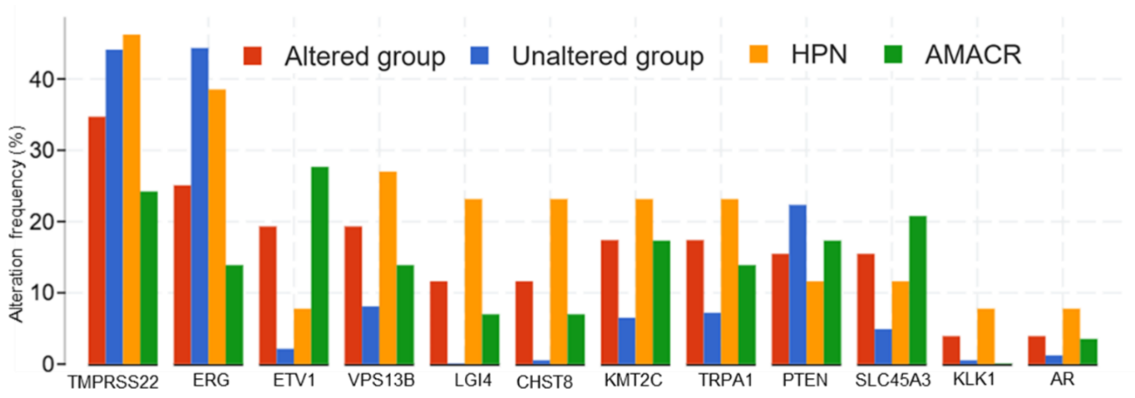

B

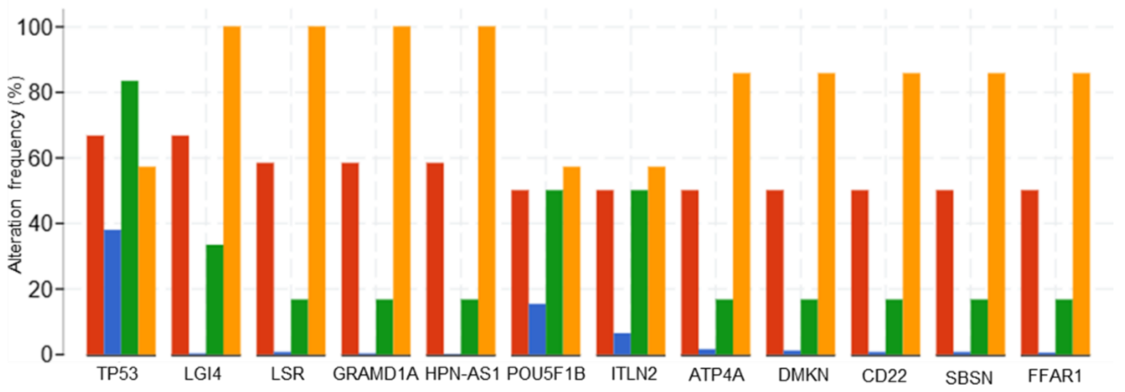

Supplementary Figure S3. Co-alteration analysis of HPN- and AMACR-associated prostate cancer datasets. (A) Frequency of co-altered prostate cancer–associated genes in tumors harboring HPN alterations within the TCGA prostate adenocarcinoma (PRAD) cohort. (B) Frequency of co-altered genes in tumors harboring AMACR alterations within the TCGA PRAD cohort. Alteration frequencies were analyzed using cBioPortal. Selected genes include prostate cancer–associated regulators and recurrently co-altered candidates identified within the analyzed datasets.
