# Supplementary material for: Phenotype-Oriented Characterization of NSC828786 Identifies Convergent HPN-AMACR-Associated Transcriptomic Signatures in Prostate Adenocarcinoma and Broad-Spectrum Antiproliferative Activity
Source: Cells. 2026 Jul 22;15(14):1314. doi: 10.3390/cells15141314 (PMC13406622; doi:10.3390/cells15141314)
Supplement: Supplementary file 1 [file cells-15-01314-s001.zip › Supplementary Fig. S4_20260717_final_revised.pdf]

Supplementary Figure S4

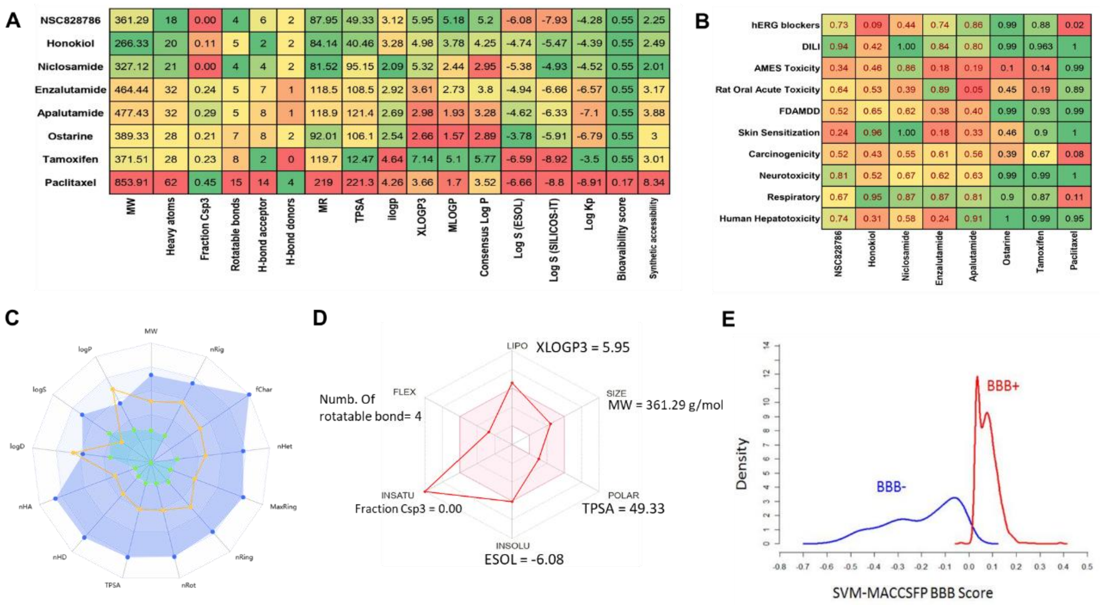

Supplementary Figure S4. In silico developability and computational pharmacologic profiling of NSC828786. (A) Comparative heatmap summarizing selected physicochemical and pharmacokinetic descriptors of NSC828786 and representative reference compounds, including molecular weight (MW), topological polar surface area (TPSA), lipophilicity-associated parameters, aqueous solubility predictions, membrane permeability, and bioavailability-related properties. (B) Computational toxicity prediction heatmap across selected safety-associated endpoints, including hERG inhibition, drug-induced liver injury (DILI), AMES mutagenicity, carcinogenicity, neurotoxicity, respiratory toxicity, skin sensitization, and human hepatotoxicity. Values represent probability- or score-based outputs generated using publicly available prediction platforms. (C) Comparative radar plot illustrating major physicochemical parameter domains of NSC828786 and representative reference compounds, including lipophilicity, molecular size, polarity, solubility, flexibility, and saturation. (D) SwissADME bioavailability radar of NSC828786, summarizing six key physicochemical properties (lipophilicity, molecular size, polarity, solubility, flexibility, and saturation) relevant to oral drug-likeness. The pink region represents the optimal physicochemical space for oral bioavailability, whereas the red polygon represents the predicted properties of NSC828786. (E) Predicted blood–brain barrier (BBB) permeability of NSC828786 generated using the SVM–MACCSFP BBB prediction model. The red and blue density curves represent the distributions of BBB-permeable

(BBB+) and BBB-impermeable (BBB−) compounds, respectively, with the predicted BBB score of NSC828786 positioned relative to these reference distributions. All results represent in silico predictions and do not constitute experimental pharmacokinetic or toxicological validation.
