# Supplementary material for: Phenotype-Oriented Characterization of NSC828786 Identifies Convergent HPN-AMACR-Associated Transcriptomic Signatures in Prostate Adenocarcinoma and Broad-Spectrum Antiproliferative Activity
Source: Cells. 2026 Jul 22;15(14):1314. doi: 10.3390/cells15141314 (PMC13406622; doi:10.3390/cells15141314)
Supplement: Supplementary file 1 [file cells-15-01314-s001.zip › Supplementary Methods_20260717_final_revised_Huang.pdf]

Supplementary Methods

Overview of computational and experimental workflow

An integrated computational and experimental workflow was applied to characterize NSC828786. Publicly available transcriptomic datasets from prostate and breast cancers were analyzed to identify differentially expressed genes (DEGs) and shared gene sets. Functional enrichment analysis and PPI network construction were performed to analyze pathway associations and identify candidate genes. Structure-based molecular docking and molecular dynamics simulations were conducted to evaluate the structural compatibility of NSC828786 with selected targets. In silico absorption, distribution, metabolism, excretion, and toxicity (ADMET) properties were predicted using established computational platforms. Experimental validation included antiproliferative profiling using the NCI-60 cancer cell line panel and developmental toxicity assessment using a zebrafish embryo model. Detailed methodological procedures are described in the following sections.

Bioinformatic analysis of GEO datasets and differential gene expression identification

Publicly available gene expression datasets were retrieved from the Gene Expression Omnibus (GEO) database [1]. Five prostate cancer datasets (GSE69223, GSE17951, GSE38242, GSE35988, and GSE55945) and three breast cancer datasets (GSE54002, GSE42568, and GSE29044), each containing tumor and adjacent normal tissue samples, were included. All datasets were accessed on March 15, 2025. Raw expression data and corresponding platform annotation files were downloaded and processed using R software (version 4.3.2). For datasets with available raw CEL files, background correction and normalization were performed using the robust multi-array average (RMA) algorithm implemented in the affy package. For datasets provided as processed expression matrices, data were log2-transformed and quantile normalized as required. Differential expression analysis between tumor and normal tissues was performed using the limma package [2]. Moderated t-tests were applied, and p-values were adjusted using the Benjamini–Hochberg false discovery rate (FDR) method [3]. Genes with an absolute log2 fold change >1.0 and an adjusted p value <0.05 were defined as differentially expressed. Volcano plots were generated to visualize expression patterns for each dataset [4]. Differentially expressed genes identified from individual datasets were intersected to obtain shared gene sets between prostate and breast cancers. Overlapping genes were visualized using Venn diagrams and used for subsequent functional enrichment and PPI network analyses [5].

Functional enrichment analysis of shared differentially expressed genes

Functional enrichment analyses were performed on the overlapping differentially expressed genes identified from prostate and breast cancer datasets. Gene Ontology (GO) enrichment analysis, including biological process (BP), cellular component (CC), and molecular function (MF) categories, as well as Kyoto Encyclopedia of Genes and Genomes (KEGG) pathway analysis, were conducted using the clusterProfiler package (version 4.8.1) in R [6-8]. Overrepresentation analysis (ORA) was performed using the enrichGO and enrichKEGG functions with Homo sapiens as the reference annotation database [9]. Multiple testing correction was applied using the Benjamini–Hochberg FDR method. GO terms and KEGG pathways with an adjusted p value < 0.05 were considered statistically significant. Enrichment results were visualized using bar plots and bubble plots generated in R. An additional enrichment analysis was conducted using the Metascape platform with default parameters unless otherwise specified [10].

PPI network analysis and genomic alteration profiling

PPI networks were constructed using the STRING database based on overlapping differentially expressed genes [11]. The analysis was restricted to Homo sapiens, and a high-confidence interaction threshold (combined score ≥ 0.70) was applied. The resulting interaction network was exported and visualized for topology analysis. Network topology parameters, including node degree and connectivity, were calculated to identify hub genes within the PPI network. Functional enrichment analysis of PPI-derived gene sets was performed using the STRING enrichment module and the WebGestalt toolkit [12]. Gene Ontology (GO; biological process, cellular component, and molecular function) and KEGG pathway analyses were conducted [13]. Statistical significance was assessed using the Benjamini–Hochberg FDR correction, with an adjusted p value < 0.05 considered significant. Genomic alteration profiles of selected candidate genes were analyzed using the cBioPortal platform [14]. Mutation frequencies, copy number alterations, and co-occurrence patterns were examined in The Cancer Genome Atlas prostate adenocarcinoma (TCGA-PRAD) and breast invasive carcinoma (TCGA-BRCA) cohorts using default platform settings.

Clinical relevance assessment and cancer hallmark annotation

Gene expression profiles were analyzed using the GEPIA2 platform [15], which integrates RNA sequencing data from The Cancer Genome Atlas (TCGA) and the Genotype-Tissue Expression (GTEx) project [16]. Expression levels were calculated as log2(TPM + 1) values, and

differences between tumor and normal tissues were assessed using default statistical parameters within the platform. Stage-specific expression patterns were evaluated using GEPIA2, where gene expression across pathological stages was compared using one-way analysis of variance (ANOVA) [17]. Survival analysis was performed using the Kaplan–Meier Plotter database [18,19]. Patients were stratified into high- and low-expression groups based on median expression values. Overall survival (OS) and relapse-free survival (RFS) were analyzed using Kaplan–Meier curves and the log-rank test. Hazard ratios (HRs) with 95% confidence intervals were calculated using Cox proportional hazards regression with default parameters. Cancer hallmark enrichment analysis was conducted using the Cancer Hallmarks Analytics Tool (CHAT) [20]. Candidate genes were mapped to hallmark categories using default platform settings.

Genomic alteration and protein expression analysis using public cancer databases

Genomic alteration profiles of candidate genes were analyzed using The Cancer Genome Atlas (TCGA) Pan-Cancer Atlas datasets accessed through the cBioPortal platform [21]. Prostate adenocarcinoma (TCGA-PRAD) and breast invasive carcinoma (TCGA-BRCA) cohorts were included. Somatic mutation frequencies, copy number alterations (GISTIC-defined amplification and deep deletion), and mRNA expression z-scores (RNA-Seq V2 RSEM; z-score threshold ±2.0) were retrieved using default study parameters. Gene-level alteration summaries and OncoPrint visualizations were generated. Protein expression levels and subcellular localization were analyzed using immunohistochemistry (IHC) data obtained from the Human Protein Atlas (HPA) database [22,23]. Staining intensity categories (high, medium, low, or not detected) and subcellular localization patterns were recorded based on HPA annotations. Representative IHC images from tumor and corresponding normal tissues were analyzed. Pan-cancer mRNA expression levels were assessed using the GEPIA2 platform [24]. Gene expression values were calculated as $\log_2(TPM + 1)$, and differences between tumor and normal tissues were evaluated using one-way analysis of variance (ANOVA) with default parameters. All database resources were accessed between March 1 and March 2, 2025.

Structure-based virtual screening, molecular docking, molecular dynamics simulation, and ADMET prediction

Structure-based molecular docking was performed to analyze the interaction of NSC828786 with AMACR and HPN. The crystal structure of HPN was obtained from the RCSB Protein Data Bank (PDB ID: 1O53). The structure of human AMACR (UniProt ID: Q9UHK6) was

obtained from the AlphaFold Protein Structure Database for structure-based analysis [25]. Ligand structures, including NSC828786 and reference compounds (bicalutamide and enzalutamide), were retrieved from the PubChem database in canonical SMILES format and converted into three-dimensional conformations using MarvinSketch (ChemAxon) [26,27]. Ligands were energy-minimized using the MMFF94 force field implemented in PyRx prior to docking. Protein structures were prepared using AutoDockTools by removing crystallographic water molecules, adding polar hydrogen atoms, assigning Kollman charges, and converting structures to PDBQT format [28]. Molecular docking simulations were conducted using AutoDock Vina with default parameters. The docking grid box was centered on the catalytic or substrate-binding regions of the target proteins. Binding affinities (kcal/mol) were calculated, and docking poses were ranked based on binding energy scores. Ligand–residue interactions, including hydrogen bonds, hydrophobic contacts, and π–π interactions, were analyzed and visualized using PyMOL. Molecular dynamics simulations were performed using standard simulation workflows under constant temperature and pressure conditions. Structural stability was evaluated based on trajectory analysis. In silico ADMET properties were predicted using publicly available web-based platforms, including SwissADME and pkCSM, to assess physicochemical properties, lipophilicity, blood–brain barrier permeability, and toxicity-related parameters [29,30].

NCI-60 antiproliferative profiling and COMPARE analysis

Antiproliferative activity data for NSC828786 were obtained from the NCI-60 human tumor cell line panel provided by the National Cancer Institute Developmental Therapeutics Program (NCI DTP). Growth inhibition data were derived from the standard five-dose in vitro screening protocol, in which compounds are tested across a defined concentration range and cellular growth is measured using the sulforhodamine B (SRB) assay. Standard response parameters, including the concentration required for 50% growth inhibition (GI_{50}), total growth inhibition (TGI), and 50% lethality (LC_{50}), were retrieved from the NCI DTP database. The NCI-60 panel comprises 60 human cancer cell lines representing multiple tissue types, including prostate (PC-3, DU-145) and breast cancer subtypes (MDA-MB-468, MDA-MB-231, MCF-7, HS-578T, T-47D, and BT-549). Response profiles of selected reference compounds, including hormone pathway modulators and cytotoxic agents, were obtained from the same database for comparison. Pattern similarity analysis was performed using the COMPARE algorithm provided by the NCI DTP. Correlation analysis was conducted using default parameters, and Pearson correlation coefficients were calculated to quantify similarity between response

patterns across the NCI-60 panel. The COMPARE gene expression module was used to analyze associations between compound sensitivity patterns and baseline gene expression profiles within the NCI-60 cell line panel [31,32].

Protein flexibility analysis using coarse-grained simulation

Protein flexibility analysis was performed using the CABS-flex 2.0 web server [33,34]. Three-dimensional protein structures in PDB format were submitted to the server, and simulations were conducted using default parameters. The resulting conformational ensembles were used to calculate residue-level root mean square fluctuation (RMSF) values, representing positional deviations of backbone residues across simulated structures. RMSF profiles were analyzed to identify regions with varying structural flexibility within the target proteins. Flexibility patterns were examined in relation to docking-predicted ligand-binding regions.

In silico pharmacokinetic, drug-likeness, and toxicity profiling

In silico pharmacokinetic and drug-likeness properties of NSC828786 were predicted using the SwissADME web server [35]. The canonical SMILES representation of the compound was submitted for analysis. Key physicochemical descriptors, including molecular weight, lipophilicity (logP), topological polar surface area (TPSA), hydrogen bond donors and acceptors, and number of rotatable bonds, were calculated. Drug-likeness was assessed according to Lipinski's Rule of Five and additional medicinal chemistry filters [36]. Gastrointestinal absorption and bioavailability scores were predicted using default settings [37]. Blood–brain barrier permeability was predicted using the BBB Predictor platform [38]. Acute toxicity was estimated using the GUSAR software package, which applies quantitative structure–activity relationship (QSAR) models to predict median lethal dose (LD_{50}) values in rodent models following oral administration. Default prediction settings were applied unless otherwise specified [39].

Zebrafish embryo-based developmental toxicity assessment

Wild-type zebrafish (Danio rerio) were maintained at 28 ± 0.5 °C under standard laboratory conditions in the Core Laboratory of Zebrafish, Taipei Medical University, in accordance with institutional animal care guidelines [40,41]. All experimental procedures were approved by the Institutional Animal Care and Use Committee (IACUC) of Taipei Medical University. NSC828786 was prepared as described in Section 2.1 [42-45]. A stock solution was prepared in DMSO and diluted in E3 embryo medium (5 mM NaCl, 0.17 mM KCl, 0.33 mM $CaCl_2$, 0.33

mM $MgSO_4$; pH 7.2) to final concentrations of 0, 5, 10, and 15 μM. The final DMSO concentration was maintained below 0.1% (v/v) in all groups. Embryos at 4 hours post-fertilization (hpf) were randomly distributed into 6-well plates (10–15 embryos per well; three independent biological replicates per concentration) and exposed to NSC828786 until 96 hpf at 28 °C. Treatment media were renewed every 24 hours. Morphological assessments were performed at 24, 48, 72, and 96 hpf using a stereomicroscope (Olympus SZX16, Olympus Corporation, Tokyo, Japan). Developmental abnormalities, including pericardial edema, yolk sac enlargement, spinal curvature, and pigmentation defects, were recorded. Quantitative morphometric measurements, including body length, yolk sac area, pericardial cavity area, and eye area, were analyzed using ImageJ software following standard calibration procedures [46,47]. Embryo viability was monitored daily. Nonviable embryos were defined by absence of heartbeat, failure of somite formation, or visible coagulation. Survival rate (%) was calculated as the proportion of viable embryos relative to the total number of embryos per group, and hatching rate (%) was defined as the percentage of embryos hatched at 72 and 96 hpf.

Statistical analysis

Statistical analyses were performed using R software (version 4.2.2), GraphPad Prism 10 (GraphPad Software, San Diego, CA, USA), and web-based bioinformatic platforms as specified in the corresponding sections. Gene expression data were $\log_2$-transformed prior to analysis. Differential gene expression analysis in GEO-derived datasets was conducted using linear models implemented in the limma package [48]. For comparisons between tumor and normal tissues in TCGA-based datasets, the Wilcoxon rank-sum test was applied. Multiple testing correction was performed using the Benjamini–Hochberg FDR method. Correlation analyses were performed using Pearson or Spearman correlation coefficients. For zebrafish morphometric measurements and survival analyses, data are presented as mean ± standard deviation (SD). Statistical comparisons among multiple groups were performed using one-way analysis of variance (ANOVA) followed by post hoc tests. For NCI-60 antiproliferative screening, percent growth inhibition values were used, and GI_{50}, TGI, and LC_{50} parameters were obtained from the NCI DTP database using standardized algorithms. All statistical tests were two-sided, and a p value < 0.05 was considered statistically significant (*p < 0.05, **p < 0.01, ***p < 0.001).

References

1. Barrett, T.; Wilhite, S.E.; Ledoux, P.; Evangelista, C.; Kim, I.F.; Tomashevsky, M.; Marshall, K.A.; Phillippy, K.H.; Sherman, P.M.; Holko, M.; et al. NCBI GEO: archive for functional genomics

data sets--update. *Nucleic Acids Res* **2013**, *41*, D991-995, doi:10.1093/nar/gks1193.
2. Ritchie, M.E.; Phipson, B.; Wu, D.; Hu, Y.; Law, C.W.; Shi, W.; Smyth, G.K. limma powers differential expression analyses for RNA-sequencing and microarray studies. *Nucleic Acids Res* **2015**, *43*, e47, doi:10.1093/nar/gkv007.
3. Kanduri, C.; Mamica, M.; Olstad, E.W.; Zucknick, M.; Li, J.J.; Sandve, G.K. Beware of counter-intuitive levels of false discoveries in datasets with strong intra-correlations. *Genome Biol* **2025**, *26*, 249, doi:10.1186/s13059-025-03734-z.
4. O'Connell, T.M. Pathway Volcano: an interactive tool for pathway guided visualization of differential expression data. *Bioinformatics* **2025**, *41*, doi:10.1093/bioinformatics/btaf367.
5. Pan, D.; Zhou, Y.; Xiao, S.; Hu, Y.; Huan, C.; Wu, Q.; Wang, X.; Pan, Q.; Liu, J.; Zhu, H. Identification of Differentially Expressed Genes and Pathways in Human Atrial Fibrillation by Bioinformatics Analysis. *Int J Gen Med* **2022**, *15*, 103-114, doi:10.2147/IJGM.S334122.
6. Yu, G.; Wang, L.G.; Han, Y.; He, Q.Y. clusterProfiler: an R package for comparing biological themes among gene clusters. *OMICS* **2012**, *16*, 284-287, doi:10.1089/omi.2011.0118.
7. Zhao, B.; Xu, Y.; Zhao, Y.; Shen, S.; Sun, Q. Identification of Potential Key Genes Associated With the Pathogenesis, Metastasis, and Prognosis of Triple-Negative Breast Cancer on the Basis of Integrated Bioinformatics Analysis. *Front Oncol* **2020**, *10*, 856, doi:10.3389/fonc.2020.00856.
8. Zhu, H.; Lin, Q.; Gao, X.; Huang, X. Identification of the hub genes associated with prostate cancer tumorigenesis. *Front Oncol* **2023**, *13*, 1168772, doi:10.3389/fonc.2023.1168772.
9. Ziemann, M.; Schroeter, B.; Bora, A. Two subtle problems with overrepresentation analysis. *Bioinform Adv* **2024**, *4*, vbae159, doi:10.1093/bioadv/vbae159.
10. Zhou, Y.; Zhou, B.; Pache, L.; Chang, M.; Khodabakhshi, A.H.; Tanaseichuk, O.; Benner, C.; Chanda, S.K. Metascape provides a biologist-oriented resource for the analysis of systems-level datasets. *Nat Commun* **2019**, *10*, 1523, doi:10.1038/s41467-019-09234-6.
11. Szklarczyk, D.; Kirsch, R.; Koutrouli, M.; Nastou, K.; Mehryary, F.; Hachilif, R.; Gable, A.L.; Fang, T.; Doncheva, N.T.; Pyysalo, S.; et al. The STRING database in 2023: protein-protein association networks and functional enrichment analyses for any sequenced genome of interest. *Nucleic Acids Res* **2023**, *51*, D638-D646, doi:10.1093/nar/gkac1000.
12. Elizarraras, J.M.; Liao, Y.; Shi, Z.; Zhu, Q.; Pico, A.R.; Zhang, B. WebGestalt 2024: faster gene set analysis and new support for metabolomics and multi-omics. *Nucleic Acids Res* **2024**, *52*, W415-W421, doi:10.1093/nar/gkae456.
13. Jin, Z.; Sato, Y.; Kawashima, M.; Kanehisa, M. KEGG tools for classification and analysis of viral proteins. *Protein Sci* **2023**, *32*, e4820, doi:10.1002/pro.4820.
14. Barnes, C.N.; Johnson, B.P.; Leacock, S.W.; Ceballos, R.M.; Hensley, L.L.; Reyna, N.S. Gene Expression and Data Analysis Pipeline Using Cancer BioPortal in the Classroom. *J Microbiol Biol Educ* **2021**, *22*, doi:10.1128/jmbe.v22i1.2315.
15. Tang, Z.; Kang, B.; Li, C.; Chen, T.; Zhang, Z. GEPIA2: an enhanced web server for large-scale expression profiling and interactive analysis. *Nucleic Acids Res* **2019**, *47*, W556-W560, doi:10.1093/nar/gkz430.
16. Chen, H.M.; MacDonald, J.A. Network analysis of TCGA and GTEx gene expression datasets for identification of trait-associated biomarkers in human cancer. *STAR Protoc* **2022**, *3*, 101168, doi:10.1016/j.xpro.2022.101168.
17. Mollah, M.M.; Jamal, R.; Mokhtar, N.M.; Harun, R.; Mollah, M.N. A Hybrid One-Way ANOVA Approach for the Robust and Efficient Estimation of Differential Gene Expression with Multiple Patterns. *PLoS One* **2015**, *10*, e0138810, doi:10.1371/journal.pone.0138810.
18. Fekete, J.T.; Gyorffy, B. ROCplot.org: Validating predictive biomarkers of chemotherapy/hormonal therapy/anti-HER2 therapy using transcriptomic data of 3,104 breast cancer patients. *Int J Cancer* **2019**, *145*, 3140-3151, doi:10.1002/ijc.32369.
19. Zhou, B.; Zhang, J.; Zhu, H.; Wu, S. A Potential Prognostic Marker PRDM1 in Pancreatic Adenocarcinoma. *J Oncol* **2022**, *2022*, 1934381, doi:10.1155/2022/1934381.

20. Menyhart, O.; Kothalawala, W.J.; Gyorffy, B. A gene set enrichment analysis for cancer hallmarks. *J Pharm Anal* **2025**, *15*, 101065, doi:10.1016/j.jpha.2024.101065.
21. Gao, J.; Aksoy, B.A.; Dogrusoz, U.; Dresdner, G.; Gross, B.; Sumer, S.O.; Sun, Y.; Jacobsen, A.; Sinha, R.; Larsson, E.; et al. Integrative analysis of complex cancer genomics and clinical profiles using the cBioPortal. *Sci Signal* **2013**, *6*, pl1, doi:10.1126/scisignal.2004088.
22. Liu, X.; Wang, J.; Chen, M.; Liu, S.; Yu, X.; Wen, F. Combining data from TCGA and GEO databases and reverse transcription quantitative PCR validation to identify gene prognostic markers in lung cancer. *Onco Targets Ther* **2019**, *12*, 709-720, doi:10.2147/OTT.S183944.
23. Uhlen, M.; Fagerberg, L.; Hallstrom, B.M.; Lindskog, C.; Oksvold, P.; Mardinoglu, A.; Sivertsson, A.; Kampf, C.; Sjostedt, E.; Asplund, A.; et al. Proteomics. Tissue-based map of the human proteome. *Science* **2015**, *347*, 1260419, doi:10.1126/science.1260419.
24. Tang, Z.; Li, C.; Kang, B.; Gao, G.; Li, C.; Zhang, Z. GEPIA: a web server for cancer and normal gene expression profiling and interactive analyses. *Nucleic Acids Res* **2017**, *45*, W98-W102, doi:10.1093/nar/gkx247.
25. Bertoni, D.; Tsenkov, M.; Magana, P.; Nair, S.; Pidruchna, I.; Querino Lima Afonso, M.; Midlik, A.; Paramval, U.; Lawal, D.; Tanweer, A.; et al. AlphaFold Protein Structure Database 2025: a redesigned interface and updated structural coverage. *Nucleic Acids Res* **2026**, *54*, D358-D362, doi:10.1093/nar/gkaf1226.
26. Pirok, G.; Mate, N.; Varga, J.; Szegezdi, J.; Vargyas, M.; Dorant, S.; Csizmadia, F. Making "real" molecules in virtual space. *J Chem Inf Model* **2006**, *46*, 563-568, doi:10.1021/ci050373p.
27. Ragno, R.; Esposito, V.; Di Mario, M.; Masiello, S.; Viscovo, M.; Cramer, R.D. Teaching and Learning Computational Drug Design: Student Investigations of 3D Quantitative Structure-Activity Relationships through Web Applications. *J Chem Educ* **2020**, *97*, 1922-1930, doi:10.1021/acs.jchemed.0c00117.
28. Arango, J.P.B.; Rodriguez, D.Y.M.; Cruz, S.L.; Ocampo, G.T. In silico evaluation of pharmacokinetic properties and molecular docking for the identification of potential anticancer compounds. *Comput Biol Chem* **2026**, *120*, 108626, doi:10.1016/j.compbiolchem.2025.108626.
29. Tang, S.L.; Sumitra, M.R.; Chen, L.C.; Liu, F.C.; Hsu, H.L.; Kuo, Y.C.; Ansar, M.; Huang, S.L.; Lee, S.Y.; Wang, H.J.; et al. Machine learning-driven discovery of NSC828779 as a multi-mechanistic NLRP3 inflammasome inhibitor for inflammatory diseases. *Comput Biol Med* **2025**, *197*, 111110, doi:10.1016/j.compbiomed.2025.111110.
30. Tang, S.L.; Sumitra, M.R.; Kuo, Y.C.; Hsu, H.L.; Ansar, M.; Huang, S.L.; Lee, S.Y.; Wang, H.J.; Lawal, B.; Wu, A.T.H.; et al. Computer-aided drug discovery of a dual-target inhibitor for ovarian cancer: therapeutic intervention targeting CDK1/TTK signaling pathway and structural insights in the NCI-60. *Comput Biol Med* **2025**, *193*, 110445, doi:10.1016/j.compbiomed.2025.110445.
31. Workman, P. The NCI-60 Human Tumor Cell Line Screen: A Catalyst for Progressive Evolution of Models for Discovery and Development of Cancer Drugs. *Cancer Res* **2023**, *83*, 3170-3173, doi:10.1158/0008-5472.CAN-23-2612.
32. Naasani, I. COMPARE Analysis, a Bioinformatic Approach to Accelerate Drug Repurposing against Covid-19 and Other Emerging Epidemics. *SLAS Discov* **2021**, *26*, 345-351, doi:10.1177/2472555220975672.
33. Kuriata, A.; Gierut, A.M.; Oleniecki, T.; Ciemny, M.P.; Kolinski, A.; Kurcinski, M.; Kmiecik, S. CABS-flex 2.0: a web server for fast simulations of flexibility of protein structures. *Nucleic Acids Res* **2018**, *46*, W338-W343, doi:10.1093/nar/gky356.
34. Kurcinski, M.; Oleniecki, T.; Ciemny, M.P.; Kuriata, A.; Kolinski, A.; Kmiecik, S. CABS-flex standalone: a simulation environment for fast modeling of protein flexibility. *Bioinformatics* **2019**, *35*, 694-695, doi:10.1093/bioinformatics/bty685.
35. Daina, A.; Michielin, O.; Zoete, V. SwissADME: a free web tool to evaluate pharmacokinetics, drug-likeness and medicinal chemistry friendliness of small molecules. *Sci Rep* **2017**, *7*, 42717,

doi:10.1038/srep42717.
36. Lipinski, C.A. Lead- and drug-like compounds: the rule-of-five revolution. *Drug Discov Today Technol* **2004**, *1*, 337-341, doi:10.1016/j.ddtec.2004.11.007.
37. Martin, Y.C. A bioavailability score. *J Med Chem* **2005**, *48*, 3164-3170, doi:10.1021/jm0492002.
38. Liu, H.; Wang, L.; Lv, M.; Pei, R.; Li, P.; Pei, Z.; Wang, Y.; Su, W.; Xie, X.Q. AlzPlatform: an Alzheimer's disease domain-specific chemogenomics knowledgebase for polypharmacology and target identification research. *J Chem Inf Model* **2014**, *54*, 1050-1060, doi:10.1021/ci500004h.
39. Lagunin, A.A.; Zakharov, A.V.; Filimonov, D.A.; Poroikov, V.V. A new approach to QSAR modelling of acute toxicity. *SAR QSAR Environ Res* **2007**, *18*, 285-298, doi:10.1080/10629360701304253.
40. Disner, G.R.; Pimentel Falcao, M.A.; Lima, C.; Lopes-Ferreira, M. Zebrafish Beyond the Bench: The 'Plataforma Zebrafish Open Doors' Programme. *Altern Lab Anim* **2021**, *49*, 175-181, doi:10.1177/02611929211057889.
41. MacRae, C.A.; Peterson, R.T. Zebrafish as tools for drug discovery. *Nat Rev Drug Discov* **2015**, *14*, 721-731, doi:10.1038/nrd4627.
42. Lee, C.C.; Chen, C.L.; Liu, F.L.; Chiou, C.Y.; Chen, T.C.; Wu, C.C.; Sun, W.H.; Chang, D.M.; Huang, H.S. Development of 1-Amino-4-(phenylamino)anthraquinone-2-sulfonate Sodium Derivatives as a New Class of Inhibitors of RANKL-Induced Osteoclastogenesis. *Arch Pharm (Weinheim)* **2016**, *349*, 342-355, doi:10.1002/ardp.201500475.
43. Lee, C.C.; Liu, F.L.; Chen, C.L.; Chen, T.C.; Chang, D.M.; Huang, H.S. Discovery of 5-(2',4'-difluorophenyl)-salicylanilides as new inhibitors of receptor activator of NF-kappaB ligand (RANKL)-induced osteoclastogenesis. *Eur J Med Chem* **2015**, *98*, 115-126, doi:10.1016/j.ejmech.2015.05.015.
44. Lee, C.C.; Liu, F.L.; Chen, C.L.; Chen, T.C.; Liu, F.C.; Ahmed Ali, A.A.; Chang, D.M.; Huang, H.S. Novel inhibitors of RANKL-induced osteoclastogenesis: Design, synthesis, and biological evaluation of 6-(2,4-difluorophenyl)-3-phenyl-2H-benzo[e][1,3]oxazine-2,4(3H)-diones. *Bioorg Med Chem* **2015**, *23*, 4522-4532, doi:10.1016/j.bmc.2015.06.007.
45. Lee, C.C.; Lo, Y.; Ho, L.J.; Lai, J.H.; Lien, S.B.; Lin, L.C.; Chen, C.L.; Chen, T.C.; Liu, F.C.; Huang, H.S. A New Application of Parallel Synthesis Strategy for Discovery of Amide-Linked Small Molecules as Potent Chondroprotective Agents in TNF-alpha-Stimulated Chondrocytes. *PLoS One* **2016**, *11*, e0149317, doi:10.1371/journal.pone.0149317.
46. Selderslaghs, I.W.; Van Rompay, A.R.; De Coen, W.; Witters, H.E. Development of a screening assay to identify teratogenic and embryotoxic chemicals using the zebrafish embryo. *Reprod Toxicol* **2009**, *28*, 308-320, doi:10.1016/j.reprotox.2009.05.004.
47. Truong, L.; Tanguay, R.L. Evaluation of Embryotoxicity Using the Zebrafish Model. *Methods Mol Biol* **2017**, *1641*, 325-333, doi:10.1007/978-1-4939-7172-5_18.
48. Lu, L.; Townsend, K.A.; Daigle, B.J., Jr. GEOlimma: differential expression analysis and feature selection using pre-existing microarray data. *BMC Bioinformatics* **2021**, *22*, 44, doi:10.1186/s12859-020-03932-5.
