# Supplementary material for: Phenotype-Oriented Characterization of NSC828786 Identifies Convergent HPN-AMACR-Associated Transcriptomic Signatures in Prostate Adenocarcinoma and Broad-Spectrum Antiproliferative Activity
Source: Cells. 2026 Jul 22;15(14):1314. doi: 10.3390/cells15141314 (PMC13406622; doi:10.3390/cells15141314)
Supplement: Supplementary file 1 [file cells-15-01314-s001.zip › Supplementary Table S1_20260527_final.pdf]

Supplementary Table S1: Growth inhibitory parameters of NSC828786 in prostate and breast cancer cell lines from the NCI-60 panel. GI_{50}, TGI, LC_{50} and IC_{50} values (µM) were obtained from the NCI DTP.

Panel	Prostate cancer		Breast cancer					
Cell Line	PC-3	DU-145	MCF7	MDA-MB-231/ATCC	HS-578T	BT-549	T-47D	MDA-MB-468
GI_{50} (µM)	1.49	1.85	1.18	0.955	1.61	1.06	0.707	1.12
TGI (µM)	>50	>50	>50	2.17	>50	7.18	-	4.35
LC_{50} (µM)	>50	>50	>50	-	>50	>50	>50	>50
IC_{50} (µM)	2.45	3.1	1.51	2.04	5.01	4.68	2.45	3.02

“ – “, indicates that the parameter was not reached within the tested concentration range. TGI not reached within the tested concentration range despite low GI_{50} values.
