# Supplementary material for: Phenotype-Oriented Characterization of NSC828786 Identifies Convergent HPN-AMACR-Associated Transcriptomic Signatures in Prostate Adenocarcinoma and Broad-Spectrum Antiproliferative Activity
Source: Cells. 2026 Jul 22;15(14):1314. doi: 10.3390/cells15141314 (PMC13406622; doi:10.3390/cells15141314)
Supplement: Supplementary file 1 [file cells-15-01314-s001.zip › Supplementary Table S2_20260717_final revised_Huang.pdf]

Supplementary Table S2: Summary of GEO microarray datasets included in differential expression analyses. Five prostate cancer (PCa) and three breast cancer (BC) cohorts containing tumor and corresponding normal tissue samples were analyzed. Platform information and sample numbers are indicated.

Accession no.	Platform	Cancer type	No. of cases	
			Normal	Tumor
GSE69223	GPL1708	PCa	16	24
GSE17951	Affy.U133Plus2	PCa	13	109
GSE38242	GPL4133	PCa	21	18
GSE55945	GPL570	PCa	8	13
GSE35988	GPL6480	PCa	12	49
GSE54002	GPL570	BC	16	417
GSE29044	GPL570	BC	36	73
GSE42568	GPL570	BC	17	104

Eight independent GEO microarray datasets were analyzed, including five prostate cancer (PCa) cohorts and three breast cancer (BC) cohorts, each comprising tumor and matched normal tissues (Supplementary Table S2). Differential expression analysis identified substantial transcriptional alterations across datasets, with DEG counts ranging from 133 to 1,160 in PCa cohorts and from 759 to 4,044 in BC cohorts, reflecting inter-cohort variability. Intersection analysis across the five PCa datasets revealed 11 consistently dysregulated genes. In contrast, cross-comparison of the three BC datasets identified 360 overlapping DEGs, indicating a broader transcriptional convergence in breast cancer. Volcano plots illustrating dataset-specific DEG distributions are shown in the following Figure. These results establish both cohort-specific variability and reproducible gene signatures within each cancer type, providing the basis for subsequent cross-cancer integrative analysis.

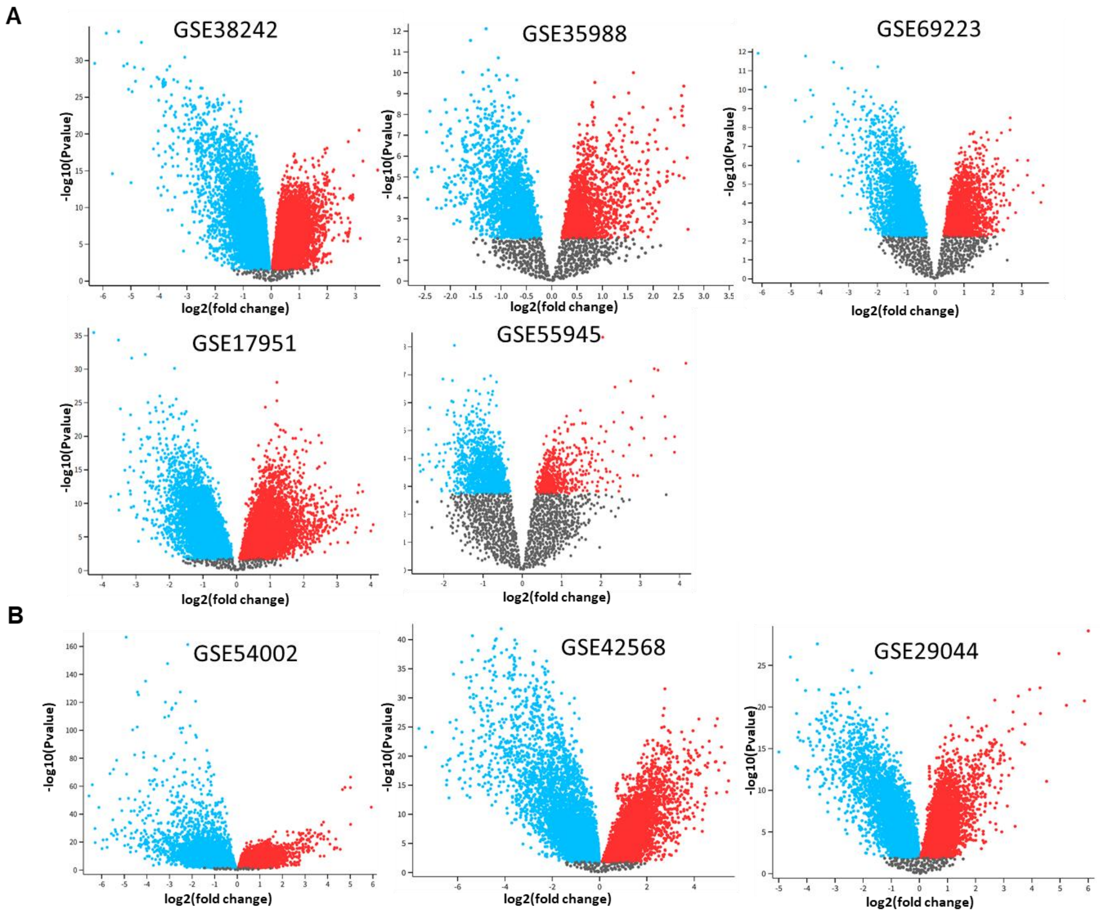

Figure. Differential gene expression and overlap analysis in prostate and breast cancer cohorts. (A) Volcano plots of DEGs identified in five prostate cancer GEO datasets. (B) Volcano plots of DEGs identified in three breast cancer GEO datasets. DEG thresholds were defined as |log2 fold change| > 1.0 and FDR-adjusted $p < 0.05$. Red: upregulated genes; blue: downregulated genes.
