# Supplementary material for: Phenotype-Oriented Characterization of NSC828786 Identifies Convergent HPN-AMACR-Associated Transcriptomic Signatures in Prostate Adenocarcinoma and Broad-Spectrum Antiproliferative Activity
Source: Cells. 2026 Jul 22;15(14):1314. doi: 10.3390/cells15141314 (PMC13406622; doi:10.3390/cells15141314)
Supplement: Supplementary file 1 [file cells-15-01314-s001.zip › Supplementary Table S3_20260528_final.pdf]

Supplementary Table S3: Predicted docking affinities and interaction profiles of NSC828786 and reference compounds with HPN and AMACR. Binding affinity values (kcal/mol) and residue-level interactions were obtained from molecular docking simulations under identical computational parameters.

Drug	Receptor	PDB ID	Binding Affinity (kcal/mol)	Key Interactions (Residues & Distances)
NSC828786	HPN	1O53	–7.5	**H-bonds:** GLY A:351 (2.08 Å), TYR A:243 (2.15 Å); **C-H:** PRO A:206 (2.54 Å), GLN A:350 (2.75 Å); **Halogen:** PRO A:245 (3.2 Å), GLN A:350 (3.68 Å), SER A:376 (3.16 Å); **π–σ:** HIS A:203 (5.43 Å); **π–alkyl:** PRO A:206 (5.18 Å).
Honokiol	HPN	1O53	–6.6	**π–σ:** LEU A:41 (3.73 Å); **alkyl:** ALA A:39 (4.28 Å, LEU A:41 (4.45 Å); **π–alkyl:** LEU A:41 (5.28 Å), TYR A:35 (5.04 Å)
Niclosamide	HPN	1O53	–7.1	**H-bond:** GLU A:207 (2.24 Å), ARG A:210 (2.44 Å), TYR A:243; **C-H:** PHE A:246 (2.72 Å); **π–σ:** HIS A:203 (5.12 Å); **π–alkyl:** PRO A:206 (4. 73Å).
Enzalutamide	HPN	1O53	–7.5	**H-bonds:** GLN A:350 (2.60Å), GLY A:351 (1.91Å), ASN A:254(2.13Å); **C-H:** GLU A:252 (3. 23Å); π–σ: HIS A:203 (4.71Å); **π–alkyl:** TRP A:377 (4. 88Å).
Apalutamide	HPN	1O53	–8.9	**H-bonds:** GLU A:207, ARG A:210; GLN A:350; GLY A:378 (2. 17-2.96Å); **π–alkyl:** TYR A:243 (5. 17Å).
NSC828786	AMACR	Q9UHK6	–7.1	**π–σ:** PHE A:27 (3.91Å); **π–alkyl:** LEU A:162 (4. 35Å), ILE A:165 (4. 82Å).
Honokiol	AMACR	Q9UHK6	–6.5	**H-bonds:** GLN A:178 (2.76), VAL A:179 (2.73); **π–sulfur:** MET A:159 (5.12); **π–σ:** ILE A:180 (3.58); **Alkyl:** MET A:166 (4.77), ALA A:167 (4. 47); **π–alkyl:** TYR A:190 (4.53)
Niclosamide	AMACR	Q9UHK6	–5.7	**π–σ:** ILE A:165 (4.24Å), ALA A:2 (3. 49Å); **π–alkyl:** LEU A:331 (4.07Å).
Enzalutamide	AMACR	Q9UHK6	–7.0	**Halogen:** H-bonds: ALA A:2 (4.07Å); **Alkyl:** LEU A:158 (4.03Å); LEU A:162 (4.3Å); **π–alkyl:** LEU A:331 (4.91Å).
Apalutamide	AMACR	Q9UHK6	–6.8	**H-bonds:** ASN A:333 (42.30Å); C-H: LEU A:162 (3.06Å); **Alkyl:** ILE A:165 (4.51Å); **π–alkyl:** PHE A:27 (4.37Å), LEU A:331 (4.83Å).

To explore potential structural compatibility with candidate targets identified from transcriptomic analyses, molecular docking simulations were performed for NSC828786 against HPN and AMACR, alongside selected reference compounds. NSC828786 exhibited comparable predicted binding affinities toward both HPN (−7.5 kcal/mol) and AMACR (−7.1 kcal/mol), suggesting a non-selective but potentially dual-compatible binding profile (Table 2). In HPN, the compound was positioned within the catalytic pocket, forming stabilizing hydrogen bonds with GLY351 and TYR243, together with additional C–H and halogen interactions involving residues such as PRO206 and GLN350 (Figure 6A–B). These interactions are consistent with the mixed polar–hydrophobic environment of the HPN active site. In AMACR, NSC828786 occupied the substrate-binding cleft and was primarily stabilized by hydrophobic interactions, including π–σ interaction with PHE27 and π–alkyl contacts with LEU162 and ILE165 (Figure 6A, C). The predominance of hydrophobic contacts is consistent with the enzyme's role in fatty acid metabolism and its ligand-binding characteristics [1]. Comparative docking analysis indicated that, although some reference compounds displayed target preference, NSC828786 maintained comparable binding scores across both proteins, supporting a potential dual-target compatibility profile rather than target-specific selectivity. These findings suggest that NSC828786 can be structurally accommodated within both proteolytic (HPN) and metabolic (AMACR) protein environments identified in transcriptomic analyses. However, these docking results represent computational predictions of binding compatibility and do not establish direct biochemical inhibition or target engagement.

References:

1. Gok, M.; Cicek, C.; Sari, S.; Bodur, E. The role of unsaturated fatty acids in modulating human butyrylcholinesterase activity: insights from kinetics and molecular docking. *Naunyn Schmiedebergs Arch Pharmacol* **2025**, *398*, 12253-12265, doi:10.1007/s00210-025-04065-3.
