# Supplementary material for: Phenotype-Oriented Characterization of NSC828786 Identifies Convergent HPN-AMACR-Associated Transcriptomic Signatures in Prostate Adenocarcinoma and Broad-Spectrum Antiproliferative Activity
Source: Cells. 2026 Jul 22;15(14):1314. doi: 10.3390/cells15141314 (PMC13406622; doi:10.3390/cells15141314)
Supplement: Supplementary file 1 [file cells-15-01314-s001.zip › Supplementary Table S4_20260528_final.pdf]

Supplementary Table S4: Predicted physicochemical and ADMET properties of NSC828786 and selected reference compounds. Physicochemical descriptors, lipophilicity parameters, pharmacokinetic predictions, drug-likeness criteria, and toxicity predictions were obtained from SwissADME and related computational platforms. Values represent in silico predictions (probability or score-based outputs depending on the model) and do not constitute experimental pharmacokinetic or toxicological validation.

Drugs	NSC	Niclosamide	Magnolol	Honokiol	Enzalutamide	Apalutamide	Tamoxifen	Paclitaxel
NSC no.	828786	178296	293099	178296	766085	771649	727681	125973
Physicochemical properties								
Formula	$C_{19}H_{11}F_4NO_5$	$C_{13}H_8Cl_2N_2O_4$	$C_{18}H_{18}O_2$	$C_{18}H_{18}O_2$	$C_{21}H_{16}F_4N_4O_2S$	$C_{21}H_{15}F_4N_5O_2S$	$C_{26}H_{29}NO$	$C_{47}H_{51}NO_{14}$
MW (g/mol)	361.29	327.12	266.33	266.33	464.44	7.43	1.5	3.9
TPSA (Å)	49.33	95.15	40.46	40.46	108.53	121.42	12.47	221.29
Molar refractivity	87.95	81.52	84.14	84.14	118.45	118.94	119.72	218.96
Rotatable bonds	4	4	5	5	5	5	8	15
H-Bond acceptors	6	4	2	2	7	8	2	14
H-Bond donors	2	2	2	2	1	1	0	4
Lipophilicity and water solubility								
iLOGP	3.12	2.09	3.28	2.96	2.92	2.69	4.64	4.26

XLOGP3	5.95	5.32	4.98	4.98	3.61	2.98	7.14	3.66
MLOGP	5.18	2.44	3.78	3.78	2.73	1.93	5.10	1.70
Consensus Log P	5.20	2.95	4.25	4.19	3.80	3.28	5.77	3.52
Log S (ESOL)	-6.08	-5.38	-4.74	-4.74	-4.94	-4.62	-6.59	-6.66
Log S (SILICOS-IT)	-7.93	-4.93	-5.47	-5.47	-6.66	-6.33	-8.92	-8.80
Pharmacokinetics								
GI absorption	High	High	High	High	High	High	Low	Low
BBB permeant	No	No	Yes	Yes	No	No	No	No
P-gp substrate	No	No	No	No	No	No	Yes	Yes
CYP1A2 inhibitor	No	Yes	Yes	Yes	No	No	No	No
CYP2C9 inhibitor	No	Yes	Yes	Yes	Yes	Yes	No	No
CYP2D6 inhibitor	No	No	Yes	No	No	No	Yes	No
CYP3A4 inhibitor	No	Yes	Yes	Yes	Yes	Yes	No	No
log Kp (cm/s)	-4.28	-4.52	-4.39	-4.39	-6.57	-7.10	-3.50	-8.91
Druglikeness								

Lipinski	Yes	Yes	Yes	Yes	Yes	No	Yes	No
Veber	Yes	Yes	Yes	Yes	Yes	No	Yes	No
Bioavailability Score	0.55	0.55	0.55	0.55	0.55	0.17	0.55	0.17
Toxicity								
AMES toxicity	0.23	0.86	0.46	0.86	0.18	0.18	0.14	0.99
hERG blocker	0.88	0.44	0.74	0.44	0.74	0.85	0.88	0.02
Hepatotoxicity	0.89	0.58	0.31	0.58	0.24	0.91	0.99	0.95
Skin sensitization	0.44	0.87	0.96	0.87	0.18	0.33	0.90	1.0

Physicochemical parameters and in silico ADMET predictions of NSC828786 were evaluated using SwissADME and related computational platforms and compared with selected reference compounds (Table 4) [1]. NSC828786 exhibited a molecular weight of 361.29 g/mol, with six hydrogen bond acceptors, two hydrogen bond donors, and four rotatable bonds, indicating a structurally moderate degree of flexibility. The calculated topological polar surface area (TPSA) of 49.33 $Å^2$ and a consensus LogP value of 5.20 suggest a relatively lipophilic profile with potential membrane permeability. Predicted aqueous solubility values from ESOL and SILICOS-IT models indicated low water solubility, consistent with its lipophilic character. Gastrointestinal absorption was predicted as "high," and the compound was not identified as a P-glycoprotein substrate, suggesting a favorable absorption profile without predicted efflux liability. Blood–brain barrier permeability predictions indicated limited central nervous system exposure (log Kp = −4.28 cm/s). Cytochrome P450 interaction predictions suggested minimal inhibitory effects on major CYP isoforms within the applied models. NSC828786 satisfied both Lipinski's Rule of Five and Veber criteria, with a predicted

bioavailability score of 0.55, indicating acceptable drug-likeness [2]. Compared with clinically used reference compounds, NSC828786 demonstrated a physicochemical and pharmacokinetic profile consistent with small-molecule oral agents, while maintaining lower molecular weight and moderate lipophilicity relative to several comparator drugs. Overall, these in silico analyses support the drug-like properties of NSC828786 and suggest that its observed antiproliferative activity is unlikely to be limited by major pharmacokinetic liabilities at the early evaluation stage.

References:

1. Daina, A.; Michielin, O.; Zoete, V. SwissADME: a free web tool to evaluate pharmacokinetics, drug-likeness and medicinal chemistry friendliness of small molecules. *Sci Rep* **2017**, *7*, 42717, doi:10.1038/srep42717.
2. Lipinski, C.A. Lead- and drug-like compounds: the rule-of-five revolution. *Drug Discov Today Technol* **2004**, *1*, 337-341, doi:10.1016/j.ddtec.2004.11.007.
