# Supplementary figures and images for: Phenotype-Oriented Characterization of NSC828786 Identifies Convergent HPN-AMACR-Associated Transcriptomic Signatures in Prostate Adenocarcinoma and Broad-Spectrum Antiproliferative Activity
Source: Cells. 2026 Jul 22;15(14):1314. doi: 10.3390/cells15141314 (PMC13406622; doi:10.3390/cells15141314)

## Slide 1
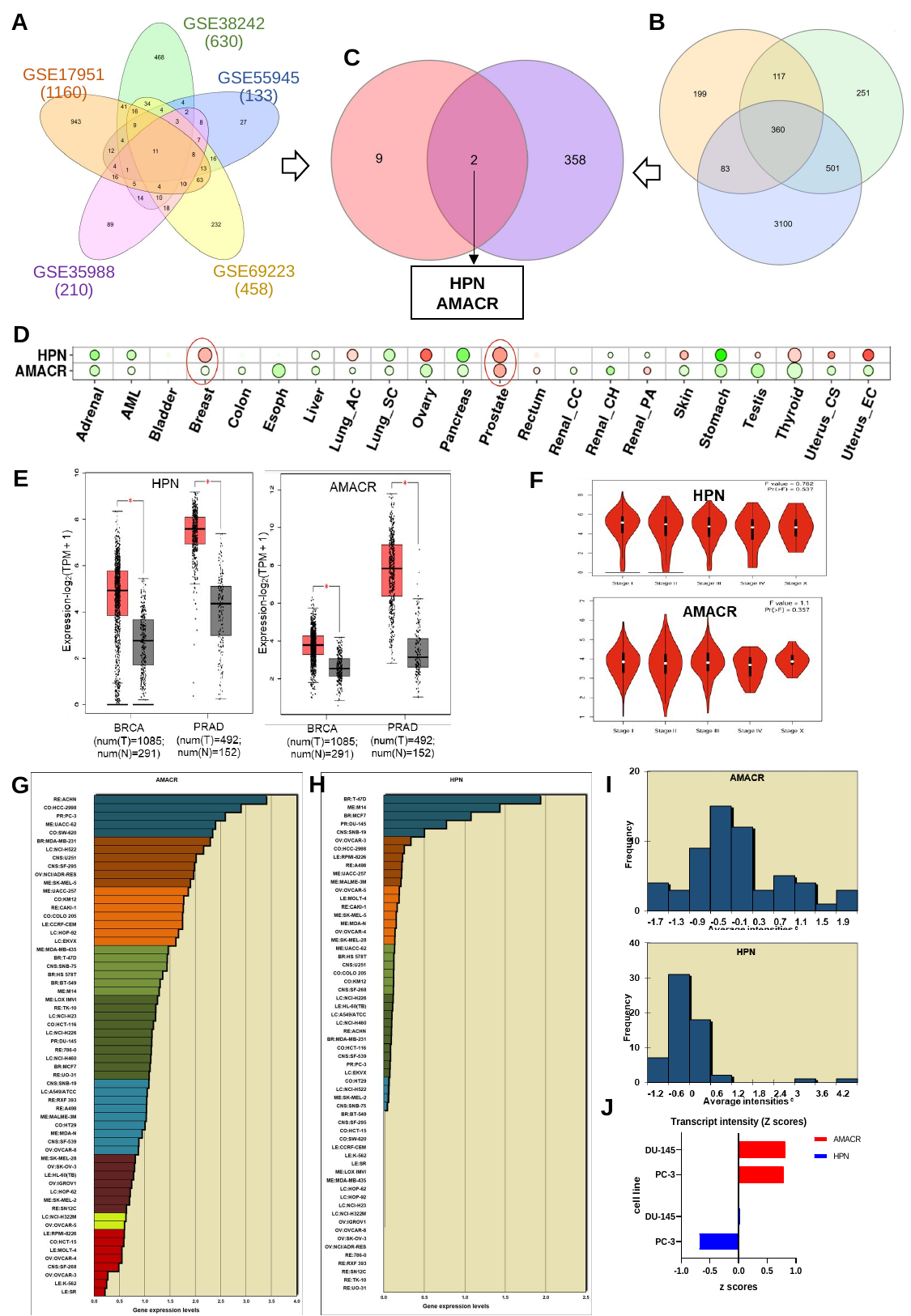

A
B
GSE38242
(630)
GSE17951
(1160)
GSE55945
(133)
GSE69223
(458)
GSE35988
(210)
C
HPN
AMACR
D
E
F
H
I
G
J

Supplement: Supplementary file 1 [file cells-15-01314-s001.zip › Supplementary Fig. S1_20260717_final.pptx]

## Slide 1
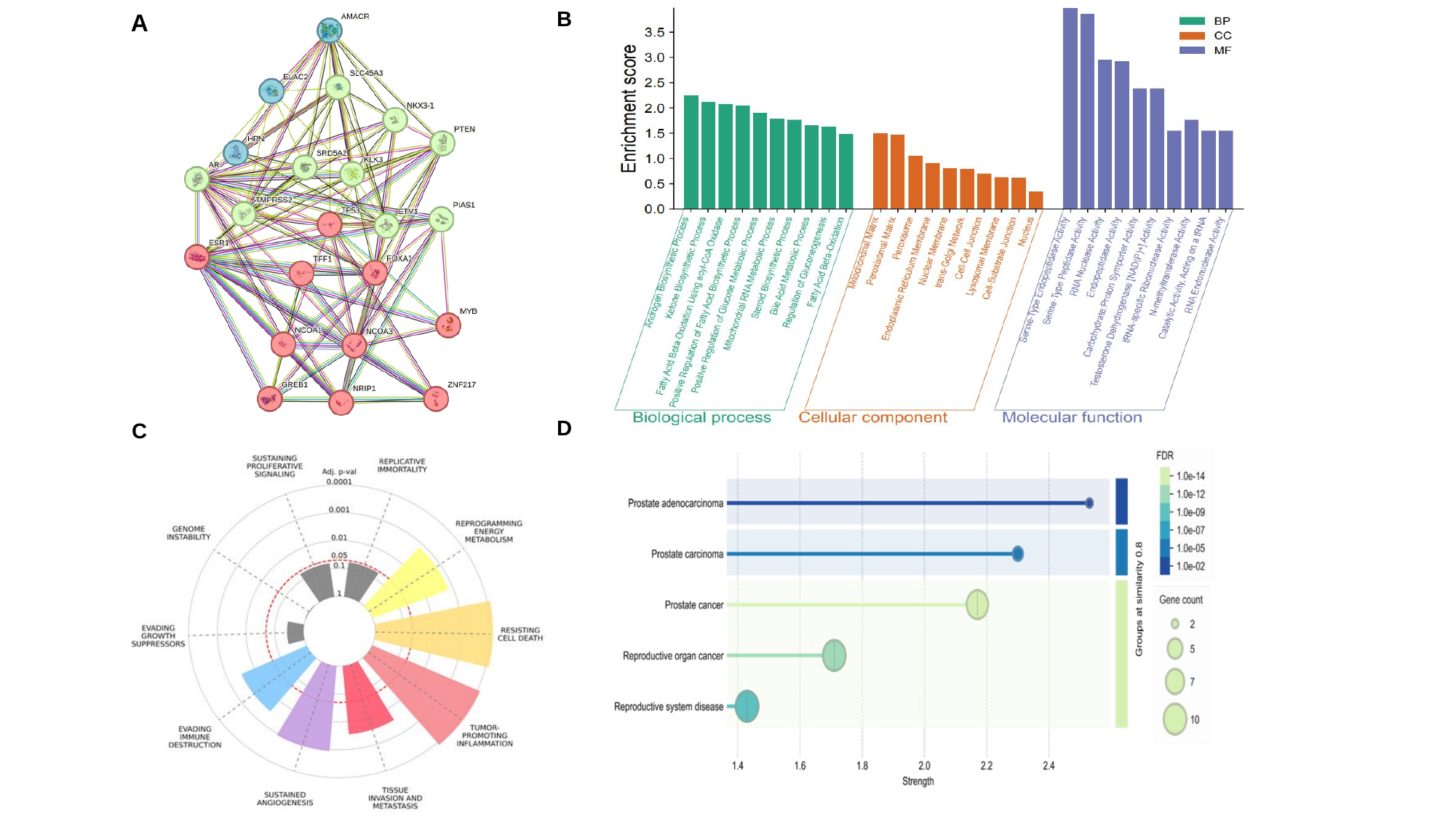

B
A
D
C

Supplement: Supplementary file 1 [file cells-15-01314-s001.zip › Supplementary Fig. S2_20260717_final.pptx]
